# Blue skies over China: The effect of pollution-control on solar power generation and revenues

**DOI:** 10.1371/journal.pone.0207028

**Published:** 2018-11-21

**Authors:** Mercè Labordena, David Neubauer, Doris Folini, Anthony Patt, Johan Lilliestam

**Affiliations:** 1 Climate Policy Research Group, Institute for Environmental Decisions, ETH Zurich, Zurich, Switzerland; 2 Atmospheric Physics Research Group, Institute for Atmospheric and Climate Science, ETH Zurich, Zurich, Switzerland; 3 Climate and Water Cycle Group, Institute for Atmospheric and Climate Science, ETH Zurich, Zurich, Switzerland; 4 Renewable Energy Policy Group, Institute for Environmental Decisions, ETH Zurich, Zurich, Switzerland; Texas A&M University, UNITED STATES

## Abstract

Air pollution is the single most important environmental health risk, causing about 7 million premature deaths annually worldwide. China is the world’s largest emitter of anthropogenic air pollutants, which causes major negative health consequences. The Chinese government has implemented several policies to reduce air pollution, with success in some but far from all sectors. In addition to the health benefits, reducing air pollution will have side-benefits, such as an increase in the electricity generated by the solar photovoltaic panels via an increase in surface solar irradiance through a reduction of haze and aerosol-impacted clouds. We use the global aerosol-climate model ECHAM6-HAM2 with the bottom-up emissions inventory from the Community Emission Data System and quantify the geographically specific increases in generation and economic revenue to the Chinese solar photovoltaic fleet as a result of reducing or eliminating air pollution from the energy, industrial, transport, and residential and commercial sectors. We find that by 2040, the gains will be substantial: the projected solar photovoltaic fleet would produce between 85–158 TWh/year of additional power in clean compared to polluted air, generating US$6.9–10.1 billion of additional annual revenues in the solar photovoltaic sector alone. Furthermore, we quantify the cost of adopting best-practice emission standards in all sectors and find that the revenue gains from the increased solar photovoltaic generation could offset up to about 13–17% of the costs of strong air pollution control measures designed to reach near-zero emissions in all sectors. Hence, reducing air pollution in China will not only have clear health benefits, but the side-effect of increased solar power generation would also offset a sizeable share of the costs of air pollution control measures.

## Introduction

Air pollution is the largest environmental cause of health damage and premature death worldwide [1]. More than 90% of the world’s population lives in places where air pollution levels surpass the limits specified by the World Health Organization (WHO) [2]. Almost every major Chinese city exceeds the limits for air pollutants recommended by the WHO, leading to some 1.1–1.6 million premature deaths annually [1, 3–5]. As a result of health impacts and forgone labor productivity, the gross domestic product (GDP) of China is decreased by up to 11% [5], and this value is increasing as China becomes more urbanized and its industrial production increases. Although reducing air pollution has clear health benefits, the monetary effects of such measures are difficult to robustly quantify, which makes arguments for expensive but effective pollution controls harder to justify. Recent aerosol modeling using satellite-derived data has quantified the effects of air pollution on surface solar irradiance [6]. Here we examine the relationship between the cost of adopting sector-specific clean-air policies and the revenues created for the solar industry from different possible regulatory mechanisms via an increase in solar generation as a result of clearer skies. We disaggregate the effects on a sectoral basis using actual anthropogenic emissions data coupled to a global aerosol-climate model. This then allows us to quantify the economic benefits associated with different clean-air policies and their costs.

## Background

Air pollution originates mainly from the burning of biofuels and fossil fuels, primarily coal. China is the world’s largest producer, consumer, and importer of coal, and it is responsible for almost half of global coal consumption [7], thereby emitting large quantities of pollutants, including sulfur dioxide (SO_2_), nitrogen oxide (NO_x_), ammonia, and carbonaceous aerosols, with large impacts on the environment and on health. To improve air quality from today’s level to non-harmful levels, it is necessary to implement aggressive clean-air policies. The Chinese central and municipal governments have implemented anti-pollution measures similar to those in industrialized economies. These include the installation and operation of pollution-control equipment on major point sources, such as coal power plants, and also on motor vehicles; the replacement of the burning of coal for residential and commercial heating with natural gas or propane; the closure of industrial plants where pollution-control equipment is not economically feasible or plants located in densely populated areas; and the gradual replacement of coal power with renewables, such as solar and wind power [8]. Although some of these policies have been successful, they do not represent the best possible outcomes: coal-burning power and industrial plants can be retrofitted with state-of-the-art pollution-control equipment to reach near-zero emissions similar to or even lower than those from a natural gas combined-cycle unit; road transport and navigation can switch to cleaner fuels with lower content of sulfur and install stricter pollution-control equipment; and residential and commercial heating and cooking can be switched from coal to natural gas nationwide [8–10]. The partial success of pollution control shows that cleaner air is possible, but it also shows that it is not easy and that the success of such policies is uncertain. In this article, we investigate the effect of pollution control on solar radiation, assuming that highly ambitious policies are implemented successfully.

To control air pollution and greenhouse gas emissions, China aims to consume 20% of its primary energy from non-fossil sources by 2030, for which renewable energy sources are crucial. China has the largest solar photovoltaic (PV) fleet worldwide [11], and the National Energy Administration recently increased the previous 2020 target of 105 GW to 200 GW [12], and the National Renewable Energy Centre foresees 400–600 GW by 2030 and 700–1300 GW by 2040 [13]. Air pollution, however, reduces the solar radiation that effectively reaches solar panels, reducing the power generation of the PV fleet [6]. Globally, this is a minor problem: on average, anthropogenic aerosol particles reduce the net radiative flux by -0.9 W/m^2^ (range from 0.1 W/m^2^ to -1.9 W/m^2^) at the top of the atmosphere [14]. However, regionally, solar dimming at the Earth’s surface can be much larger, as vividly evidenced during smog events around the world. Air pollution affects solar power generation through three main mechanisms. First, particle matter accumulates on the solar panels [15], which reduces generation until the panels are washed. Second, aerosol particles such as sulfate, black carbon (BC), organic carbon (OC), and sea salt or dust particles, interact in ways that scatter (and sometimes absorb) solar radiation [14]. Third, cloud formation caused for example by the reaction of SO_2_ with other pollutants, creating aerosol sulfate particles, absorbs moisture from the air and can serve as cloud condensation nuclei (certain aerosol particles also as ice nuclei), thereby increasing cloud reflectivity [16] and lifetime [17] and decreasing the solar radiation reaching the Earth’s surface.

Most industrialized countries, including those of the European Union and the United States, have adopted stringent air quality standards, primarily out of a concern for the human health impacts of ground-level pollution, but also causing substantial economic cost [18]. China has been moving in this direction, but nevertheless lags behind in terms of air quality. Given the ministerial decision-making structure of the Chinese government, there is reason to believe that an appraisal of the costs and benefits lying entirely within the Chinese energy system, of such pollution control policies, could provide a politically salient argument for accelerating the improvements in Chinese air pollution.

## Materials and methods

We perform a cost-benefit analysis to compare the cost of the measures on the fossil-fuel sectors to reach near-zero emissions with the increase in revenues created for the solar industry. We do this in three steps.

First, we estimate the cost of implementing clean-air policies on various fossil-fuel sectors, namely the energy, industrial, residential and commercial, and transport sectors. For each sector, we estimate the cost of implementing sector-specific best-practice emission standards and apply the cost functions to sector-specific emissions. The emission data are from the bottom-up emissions inventory Community Emission Data System (CEDS) [19].

Second, we model the effect of eliminating anthropogenic emissions on surface solar irradiance with the global aerosol-climate model ECHAM6.3-HAM2.3 and disaggregate the effects of different pollution control measures in each sector. To estimate the effect of emissions on surface solar irradiance, we estimate both the direct effect of aerosol particles (scattering and absorption) on solar radiation and the semi- and indirect-effects of aerosol particles on cloud formation and life-time. We acknowledge that emissions in the future can change, and indeed that is a prerequisite for our study: the Chinese policies have strongly reduced emissions from power stations, and the next steps can be to strongly reduce industrial, commercial and transport-related emissions.

**Fig 1:**
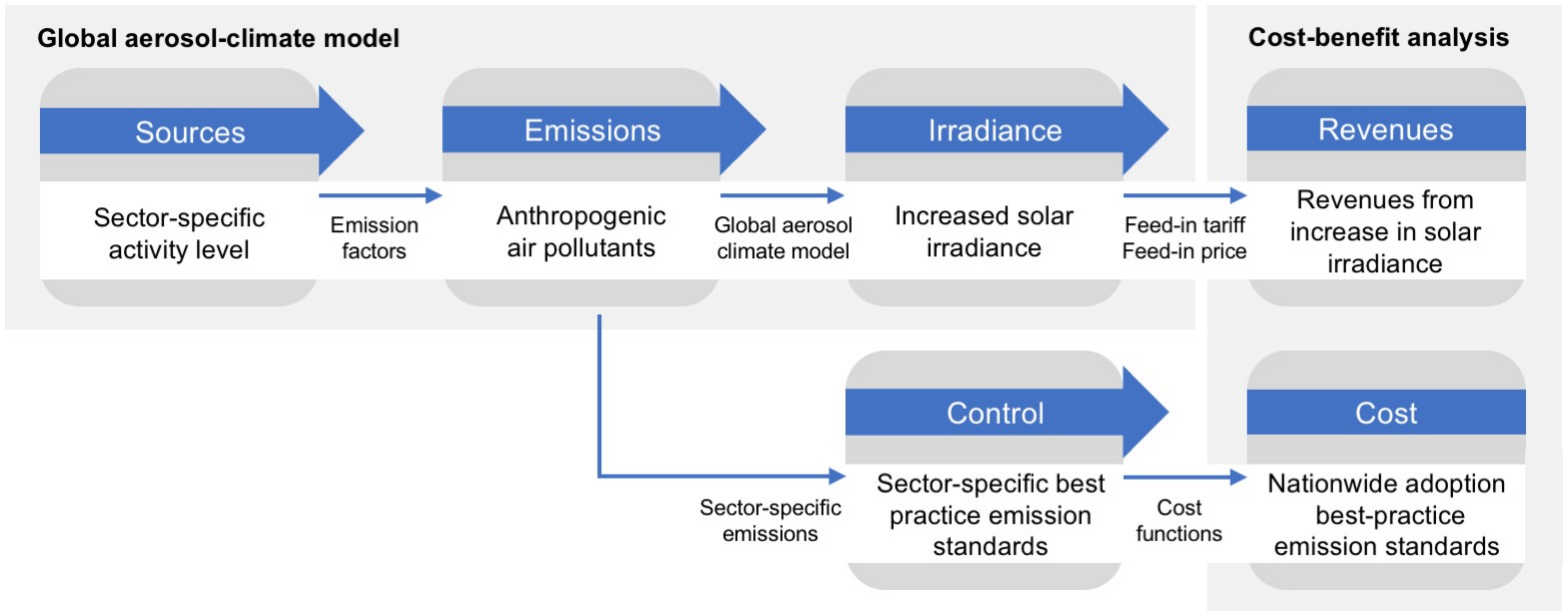
Framework for evaluating the cost of clean-air policies and the revenues to the solar industry.

Third, we estimate the increase in electricity generation from solar PV panels as a result of an increased surface solar irradiance due to a reduction in emissions and calculate the revenues for the solar industry from the current market feed-in tariffs (FiTs) and future feed-in prices into the electricity grid. We discount the future revenues for two different discount rates to account for various discounting strategies in the private sector. Fig 1 introduces the framework with which to estimate the cost of adopting best-practice emission standards and the economic gains to the solar industry from clean air.

To perform the cost-benefit analysis we use the following net present value (NPV) model:

$$NPV=\sum \;[\frac{{Cost}_{n}-{Revenues}_{n}}{{\left(1+r\right)}^{n}}]$$ (1)

where *Cost* is the cost incurred to investors as a result of adopting best-practice emission standards (described in the following section *Clean-air policies and their cost*), *Revenues* is the revenues to the solar investors from the feed-in tariffs (FiT) and feed-in price from increased solar irradiance (described in section *Revenues to solar investors*), *n* the number of years between 2016 and the target years, and *r* the discount rate.

### Clean-air policies and their cost

Here we briefly introduce the air pollution control measures on current fossil-fueled installed capacity and transport fleet. See section 1 in S1 Supporting Information for a detailed description of the methods and calculations for the estimation of the sector-specific costs to adopt best-practice emission standards. We assume that future assets will be near-zero emitters as a result of new standards and hence, reaching near-zero emissions for these assets comes at no additional cost.

#### Electricity generation

China is leading an ambitious, multi-front campaign to clean up the air [8]. In the power sector, China is mothballing older coal-fired power plants and reducing emissions from existing plants by retrofitting them with air pollution control technologies [8]. Several coal-fired power plants have become early adopters of near-zero emission control technologies, resulting in emissions that are lower than the most stringent regulation limits for coal plants and also below the limits for natural gas turbine plants: PM 5 mg/m^3^, SO_2_ 35 mg/m^3^, and NO_x_ 50 mg/m^3^ [9]. Here we assume that combustion processes in fossil-fueled power plants are equipped with emission-removal technologies to achieve near-zero emissions.

The combustion process for heat generation due to electric boilers that generate heat for sale to third parties, such as residential, commercial, or industrial consumers, can be equipped with similar air pollution control systems as is used in fossil-fueled power plants [20]. The same applies to the transformation processes, including coal coke production, oil refining, and charcoal production. We estimate that the total retrofitting cost in the energy sector, i.e., electricity and heat generation plants and transformation processes, is US$7.2–11.4 billion/year.

#### Industrial combustion and industrial processes

Although emissions from coal-fired power plants and coal-fired industrial boilers are affected by a number of variables, such as coal type and composition and the type of combustion technology, the emission control technologies used to limit emissions of flue gases are similar [20]. Hence, we assume that coal-fired industrial boilers are retrofitted with the same combination of air pollution control systems as is used in coal-fired power plants, and thus the total cost is US$12.0–19.1 billion/year.

In addition to the emissions from combustion processes in industry, the sector emits process emissions that occur (a) as a result of the thermal decomposition of substances, (b) of reactions between substances or their transformation, such as the chemical or electrolytic reduction of metal ores, and (c) during the creation of substances for use as feedstock [21]. In China, most of the process emissions originate from the metal production industry, the chemical industry, and the pulp and paper industry. Emission control technologies for process emissions are process-specific and strongly dependent on the quality of raw materials. The literature available on the costs to limit processes’ emissions is limited. Hence, we acknowledge some uncertainty regarding the costs to limit these emissions, which account for ~15% of total SO_2_ emissions, the pollutant with the highest influence on reducing solar surface irradiance. We thus estimate that the cost of retrofitting the industrial processes is US$4.2–6.7 billion/year.

#### Road transport and domestic navigation

We estimate the cost of reaching near-zero emissions from road transport and domestic navigation by switching to near-zero-sulfur 10 ppm (parts per million) fuels. The costs of reducing sulfur content in the fuel depend on the state of existing refineries, current fuel quality, and emissions standards but such costs can be divided into two types: the cost associated with fuel production and the cost associated with vehicle emission control technologies. Estimates of the costs associated with fuel production accounts for upfront refinery investment, such as capital equipment upgrades, and direct operating costs, such as catalysts and chemicals [10]. Estimates of the cost for the introduction of advanced emission control technologies in vehicles account for the additional costs to manufacturers for equipping these vehicles with advanced emission control technologies to meet international best-practice standards, i.e., the adoption of the China 6 standard in gasoline and diesel vehicles. The adoption of international best-practice standards such as ultra-low sulfur standards and the China 6 standard costs US$11.7 billion/year.

For marine vessels, switching from high-sulfur heavy fuel oil (HSFO) to low-sulfur marine diesel or gas oil (MDO/MGO) is a straightforward solution because engines do not need to be retrofitted with emission control technologies to accept this type of fuel, although minor adjustments in auxiliary equipment are needed in some cases. We estimate the cost of retrofitting the oceangoing container vessels in Chinese waterways to use MDO/MGO, i.e., installing a fuel cooler or chiller and the associated piping prior to the fuel pump to decrease fuel viscosity, and selective catalytic reduction (SCR) technology. The total cost of retrofitting the container fleet is US$3.6 billion/year. We exclude emissions from rail transport because these account for only 3% of SO_2_ emissions from the transport sector alone.

#### Residential and commercial sector

To improve air quality, China has started to replace coal-fired residential heating and cooking in northern Chinese cities with gas-powered stoves and boilers, or with those using electricity from renewable energy [8]. We estimate the cost of replacing coal with gas from natural gas pipelines and liquefied natural gas imports for the residential and commercial sector. Switching from coal to gas involves the construction of natural gas distribution networks, pipelines, and household connection facilities, the prices of which are uncertain. The data on the installed capacity of residential and commercial boilers, and the cost of converting a coal-fired boiler to a natural gas-fired boiler are also uncertain. Hence, we acknowledge these uncertainties and exclude these estimates from our calculations. We estimate that the cost of replacing coal with natural gas for residential and commercial use is US$9.9–16.1 billion/year.

### Effect of clean-air policies on surface solar irradiance

We construct several emission scenarios that replicate the policy changes driving emission reductions, and quantify the geographically specific changes in surface solar irradiance as a result of reducing or eliminating sector-specific air pollutants. Reference [6] quantified the impact of aerosols on surface solar irradiance in China using satellite observations, which account for all aerosols. Aerosols, however, can be of anthropogenic (e.g., power plants, slash-and-burn agriculture, incinerators, cooking stoves, and vehicles) or natural origin (e.g., volcanoes, dust storms, and forest fires). In estimating the impact of aerosols from anthropogenic sources only, satellite observations are uncertain data sources, because the aerosol origin cannot be easily determined, making it difficult or impossible to assess the impacts of sector-specific measures using satellite data. Some studies have attempted to identify aerosol origin from a combination of satellite observations and models [22, 23] but these only differentiate between anthropogenic and natural aerosol origin and provide no sector-specific data. Here, in contrast to [6], we use region- and sector-specific emission data to model the effect of anthropogenic emissions on surface solar irradiance using a bottom-up approach and disaggregate the effects of current emissions and of pollution control measures in each sector.

To examine the effect of anthropogenic aerosols on surface solar irradiance, we use the global aerosol-climate model ECHAM6-HAM2, which consists of the global climate model ECHAM6 [24] and the aerosol module HAM2 [25, 26]. The global aerosol-climate model ECHAM6-HAM2 allows us to identify the impact of single anthropogenic aerosol species and examine sector-specific pollution control scenarios. Here, we use the latest model version, ECHAM6.3-HAM2.3, and calculate the direct effect of aerosol particles (scattering and absorption) on solar radiation and the semi- and indirect-effects of aerosol particles on cloud formation and life-time.

ECHAM6.3-HAM2.3 has a two-moment-cloud microphysics scheme [27, 28] to compute the interactions of aerosol particles with stratiform liquid, mixed-phase, and ice clouds. The interactions of aerosol particles with convective clouds are not explicitly included, but the convection scheme ([29], with modifications by Nordeng [30] for deep convection) uses the dependence of the detrained cloud droplets (i.e., for liquid clouds only) from convective clouds, on number concentration of activated cloud condensation nuclei at the base of the convective clouds.

In the climate model ECHAM6.3, we compute the radiative transfer using the broadband radiative transfer model Psrad [31]. Psrad uses 14 bands for the shortwave and 16 bands for the longwave part of the spectrum. In the aerosol module HAM2.3, we use a sulfur chemistry module based on Feichter, Kjellstrom [32]. The aerosol module computes the life cycle of aerosol particles: the emissions of precursor gases or aerosol particles, the nucleation of new sulfate aerosol particles and condensation of gaseous sulfuric acid on existing aerosol particles, aerosol particle collisions and growth, the water uptake of aerosol particles, the interactions of aerosol particles with radiation and clouds, and the removal of aerosol particles by sedimentation, dry deposition, wet scavenging in clouds, or precipitation below clouds. The aerosol module uses the aerosol species of sulfate, BC, OC, sea salt, and mineral dust particles. Sulfate is computed from SO_2_ and dimethyl sulfide emissions. Natural aerosol emissions of sea salt and mineral dust and dimethyl sulfide precursor emissions from the oceans are computed online. Anthropogenic SO_2_, BC and particulate organic matter are taken from CEDS for the last available year of data, which is 2014. Observed sea surface temperatures and sea ice cover are for the years 2000–2009 (AMIP simulations). Meteorological variables, including vorticity, divergence, and surface pressure were nudged towards the ERA-Interim reanalysis for the same years [33]. The model does not compute the impact of climate change on clouds.

We conducted several 10-year simulations (after a 3-month spin-up), varying the emissions from the different sectors in China only. All simulations were performed with a T63 horizontal spectral resolution of 1.9°×1.9° using 31 vertical levels.

The sum over the shortwave bands is the shortwave downward flux at the surface. Here, we refer to it as surface solar irradiance. We estimate the increase in surface solar irradiance by subtracting the modelled surface solar irradiance under certain emissions abatement conditions from the surface solar irradiance under current or specific abatement emissions conditions. Because surface solar irradiance depends nonlinearly on the aerosol-radiation and aerosol-cloud interactions (Figure 3 in Carslaw, Lee [34]), it increases further the stronger the emissions abatement, by following a logarithmic growth pattern.

Differences in calculating surface solar irradiance with ECHAM6.3-HAM2.3 and satellite observations such as CERES-SYN1deg, as used in [6], may appear. This may result from the fact that the version of the CERES-SYN1deg used by [6] excludes, in the *no aerosol* product, the total aerosol optical depth (i.e. also the aerosol optical depth from background aerosol such as mineral dust, sea salt aerosol, other marine aerosols, biomass burning aerosol, aerosol from vegetation). In ECHAM6-HAM2, background aerosol data are included in all simulations. Differences in surface solar irradiance from ECHAM6.3-HAM2.3 may also appear when compared to surface observations. See section 2 in S1 Supporting Information for an evaluation of the modeled surface solar irradiance with ECHAM6.3-HAM2.3 compared to satellite observations from CERES-SYN1deg and surface observation-based estimates.

ECHAM6.3-HAM2.3 does not account for nitrate aerosol and CEDS does not provide data on anthropogenic dust; thus, the results presented in the section *Results and discussion* on reductions in surface solar irradiance are rather an underestimation of what solar irradiance could further be reduced, thereby possibly underestimating the potential additional revenues to the solar industry.

We examine the effect of past and future decisions of reducing or eliminating emissions on surface solar irradiance. For this, we model the effect of reducing sector-specific emissions from past and counterfactual scenarios and the effect of eliminating sector-specific actual emissions to account for future measures.

**Fig 2:**
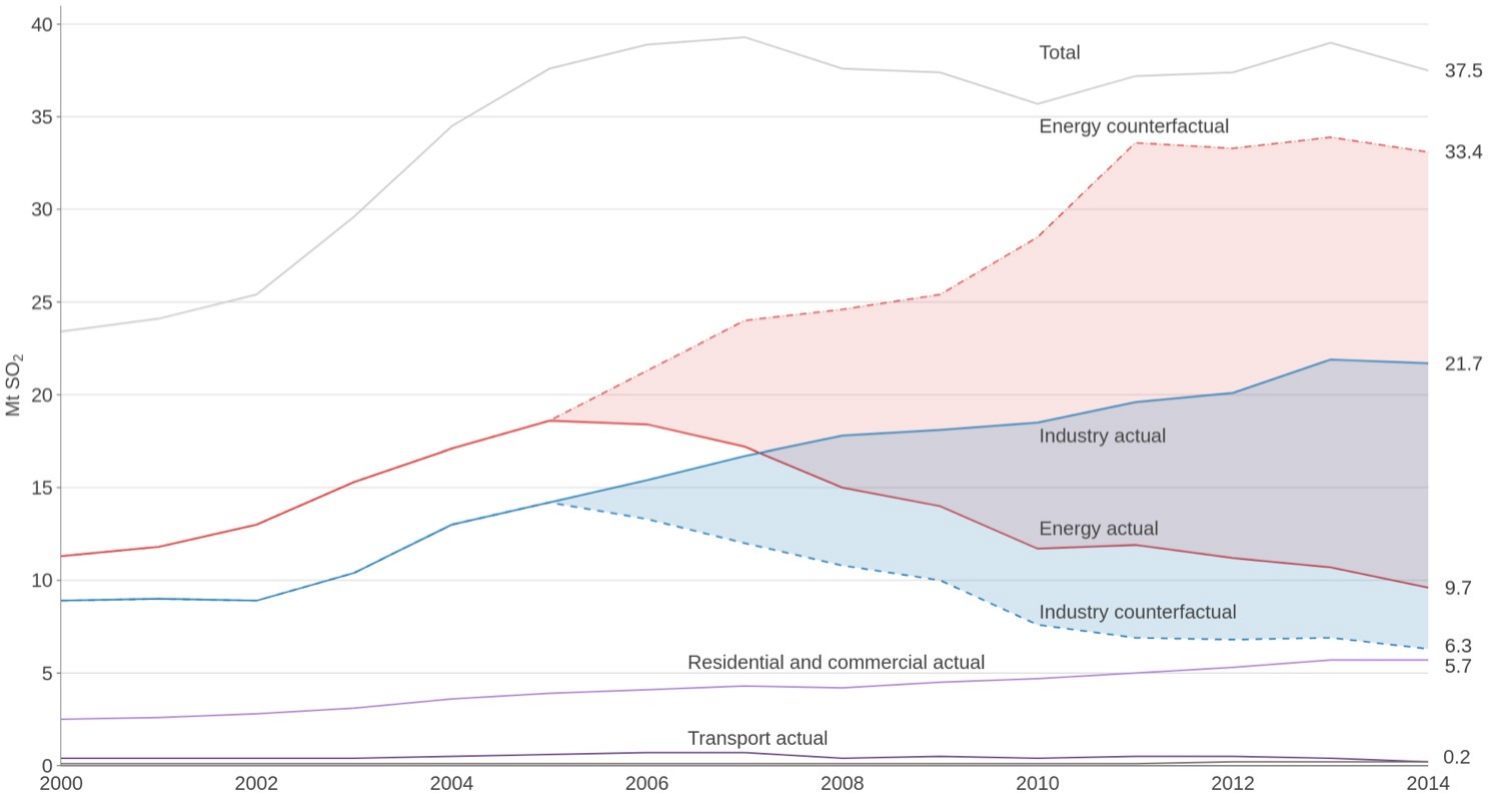
Yearly SO_2_ emissions (Unit: Megatonnes, Mt) in China. Emissions from energy generation, industry, residential and commercial, and transport sectors based on the bottom-up inventory of anthropogenic emissions Community Emission Data System [19]. Solid lines are actual emissions; the dashed ones are the counterfactual scenarios. The four sectors account for 99% of SO_2_ emissions; the remainder comes mainly from waste incineration.

Fig 2 shows the actual and counterfactual SO_2_ emissions from the energy, industrial, residential and commercial, and transport sectors. The counter-factual scenarios describe the “could-have-been” emissions if no emission standards had been implemented since 2006 for the energy sector and the emissions of the industrial sector if the same standards used for electricity generation had been applied to industry as well (section 3 in S1 Supporting Information for a description of policies and measures adopted in the past to control air pollution; and section 4 in S1 Supporting Information for details on the calculation of the counterfactual scenarios). As seen in Fig 2, the emission standards in the energy sector, mainly thermal power plants for electricity production, worked well: emissions decreased by about 50%, even though coal power generation increased dramatically. In the industrial sector, which includes iron and steel, cement, non-ferrous metal smelting, the chemical industry, and other industry boilers, the SO_2_ control measures are much weaker, and emissions are steadily increasing. The emissions of the residential and commercial sectors, mainly from heating, are slowly increasing, and there is no coordinated policy to control these emissions. Table D in S1 Supporting Information shows the sector-specific contributions of anthropogenic SO_2_, BC and OC emissions as estimated by CEDS, the latest sector-specific emissions data available.

### Revenues to solar investors

**Fig 3:**
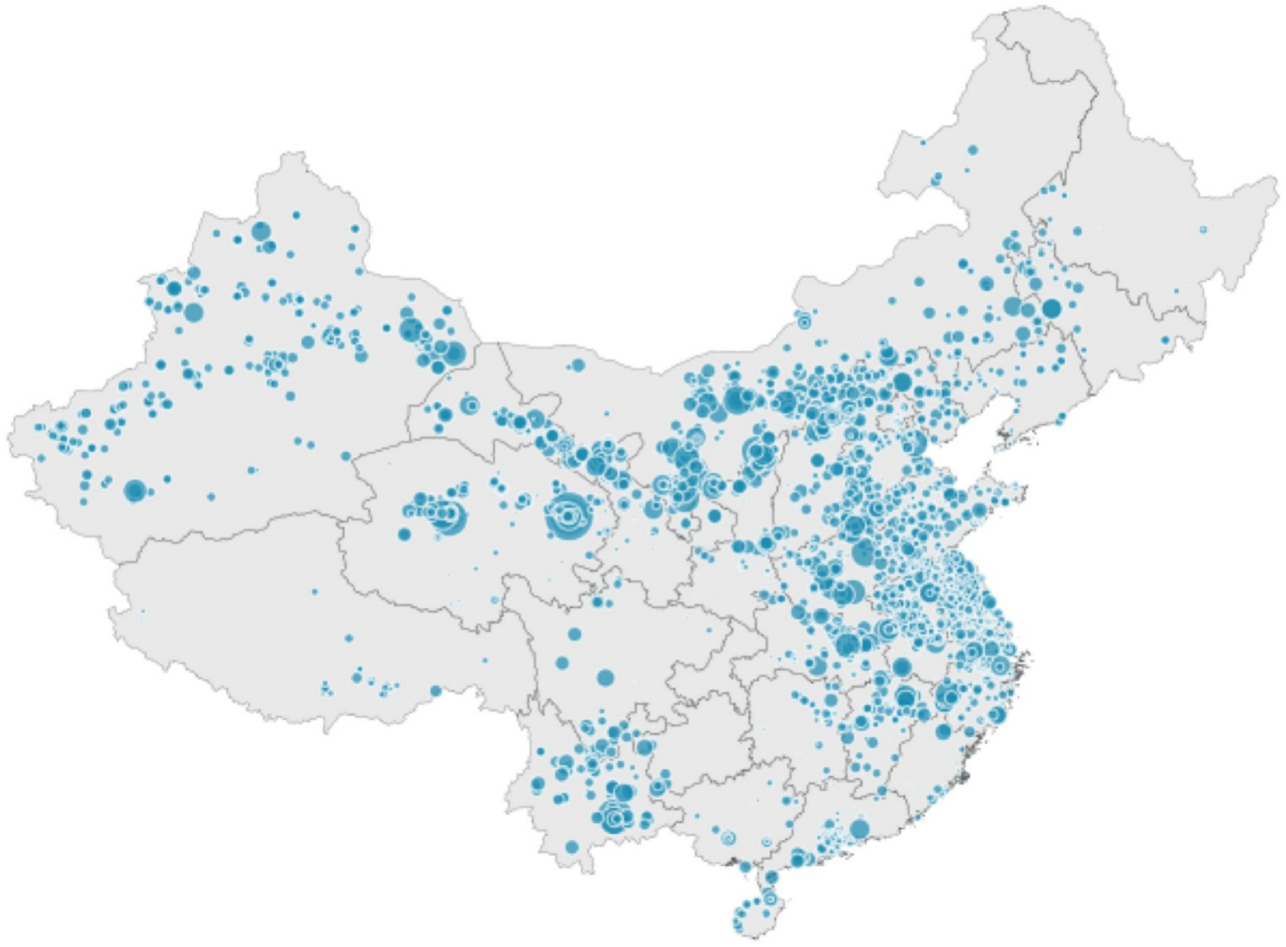
Distribution of the grid-connected solar PV plants scaled by capacity. Data from Bloomberg NEF [11] under a CC BY license, with permission from [11].

We estimate the increase in electricity generation from solar PV panels using the modelled net surface solar irradiance from ECHAM6.3-HAM2.3. The increased revenue is thus the difference in income from electricity generation of all solar panels under actual conditions and under the emissions conditions of a specific scenario. We classify the operational grid-connected solar PV installations in China as of December 2016 depending on their locations (for a given latitude and longitude), installed capacities, and operation dates, as given by Bloomberg New Energy Finance [11] (see Table E in S1 Supporting Information for cumulative installed solar PV capacities by province and region). Fig 3 shows the distribution of the grid-connected solar PV plants scaled by installed capacity. Bloomberg New Energy Finance does not provide data on the location and capacity of non-grid-connected solar PV projects; hence, we exclude these projects from the remuneration calculation in this analysis.

We compute the annual electricity generation E (kWh) from a given fixed (non-tracking) solar PV system as follows:

$$E=A\;r\;I_{tr}\;\eta \;h=P_{dc0}\;I_{tr}\;\eta \;h$$ (2)

where *A* is the total solar panel area (m^2^); *r* is the solar panel efficiency (%); *I*_*tr*_ is the increase in surface solar irradiance (kW/m^2^); *η* is the system performance ratio, which we assume as a uniform 0.85 [35]; *h* are hours in a year, 8760; and *P*_*dc0*_ is the nameplate DC rating of the module (kW). We apply a correction factor to the electricity generated to account for optimal panel orientation and tilt based on JRC [36], see Table F in S1 Supporting Information. We calculate the annual electricity generation from 2016 to 2040 for an installed PV capacity of 78 GW in 2016 [11] and future PV capacities according to different scenarios (200, 400–600, and 700–1300 GW by 2020, 2030, and 2040, respectively) [12, 13]. The International Energy Agency projects 469–550 GW of PV capacity by 2030 and 738–835 GW by 2040 [37], in agreement with [13]. We assume a linear capacity-growth trend from current installed capacities to future capacity scenarios based on current provincial capacity distribution.

We then calculate the revenues for the solar industry from the current market FiT and future feed-in prices into the electricity grid. To calculate the potential remuneration from each grid-connected PV project, we multiply the project-specific annual electricity generation by the region-specific FiT scheme. We do so for all operational grid-connected solar PV projects and calculate the total remuneration. See section 8 in S1 Supporting Information for information on the compensation level of the FiTs depending on the region and starting date of the compensation.

It is expected that the FiTs for new PV projects will decrease over time to reflect decreasing technology costs. We assume that the FiT level, or eventually the feed-in price once the tariff is removed, follows the national utility-scale PV system cost, which was 1,168 US$/kW in 2016 [38], for two different scenarios. First, we assume that FiTs decrease over time, following a decrease in PV cost under a technological learning rate of 20% starting from 2017, the last year of available FiT data (Table G in S1 Supporting Information). See Eq (3) for a description of the learning curve in terms of how the system cost evolves over time [39].

$$C_{cum}=C_{0}\;n^{b}$$ (3)

where *C*_*cum*_ is the cost per unit as a function of cumulative capacity, *C*_*0*_ is the cost of the first unit, *n* is the cumulative capacity, and *b* is the experience index. The costs decrease by the learning rate LR = 1–2^b^ for each doubling of cumulative capacity.

In the second case, we assume that no technological learning occurs after 2017, so that both the technology cost and the FiTs are the same as in 2017.

We estimate future system costs given an actual (78 GW by the end of 2016) [11] and future PV capacities as expected by the National Energy Administration and the National Renewable Energy Centre (200, 400–600, and 700–1300 GW by 2020, 2030, and 2040, respectively) [12, 13]. We exclude the revenues from off-grid solar PV installations when estimating the revenues. We discount the revenues to the solar investors using discount rates of 5% and of 8%, which are commonly used on mitigation options in China [10, 38].

## Results and discussion

### Cost of air pollution measures for near-zero emissions

The cost of adopting sector-specific nationwide clean-air policies to reach near-zero emissions, for the present market configuration, amounts to US$58.6 billion/year, ±US$10 billion/year (Table I in S1 Supporting Information). The highest cost of pollution control stems from industry because this is the largest polluter, but the specific cost of pollution elimination is different in each sector and also for each pollutant because the processes differ widely. Monetary values are in US$_2016_/kW, exchange rate 0.151 of annual average RMB_2016_.

### Effect of clean-air policies on surface solar irradiance: Nationwide

**Fig 4:**
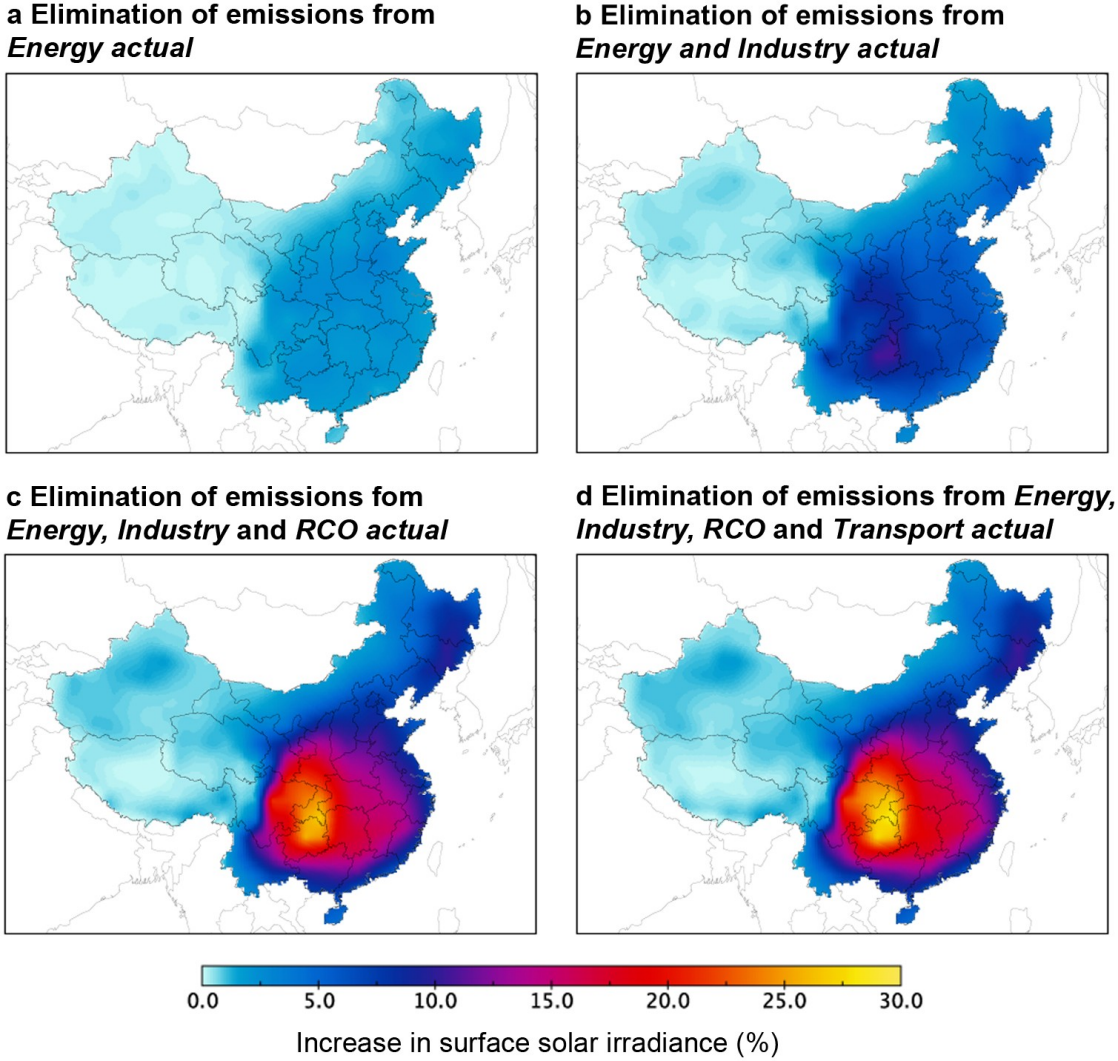
Increase in surface solar irradiance in percent (%). From an elimination of actual SO_2_, BC, and OC emissions from energy sector (a), of actual emissions from energy and industrial sectors (b), of actual emissions from energy, industrial and residential and commercial (RCO) sectors (c), and of actual emissions from energy, industrial, RCO, and transport sectors (d). Data and material from [19, 24–26].

Past air pollution control measures have led to an increase in solar irradiation: compared to the counter-factual emissions levels without pollution control, the air pollution control policies implemented in the energy sector since 2006 have increased surface solar irradiance by up to 3.5% (5 W/m^2^) (section 10.1 *Effect of past and counterfactual clean-air policies on surface solar irradiance* in S1 Supporting Information; Figs B and C in S1 Supporting Information for % and W/m^2^ results). Yet, there is still room for further improvement considering today’s pollution levels. Here we show the effect of decisions to eliminate sector-specific emissions on surface solar irradiance. Results show that the increase in surface solar irradiance due to emissions reduction is non-linear: eliminating today’s emissions from the energy sector increases surface radiation by up to 3.5% and eliminating industry emissions as well increases radiation by up to 11% (Fig 4a and 4b; 6–16 W/m^2^, Fig D in S1 Supporting Information for W/m^2^ results). Eliminating the emissions originating in the residential and commercial (RCO) sector as well, however, increases surface solar irradiance by up to 27% (Fig 4c; 35 W/m^2^, Fig Dc in S1 Supporting Information). The contribution of emissions from transport is small as compared to those of the other sectors, and removing its emissions as well increases surface solar irradiance only by up to 29%, representing only a slight increase (Fig 4d). Hence, whereas the effects of eliminating emissions in a single sector are small (5% from industry only, 3% from residential and commercial only, Fig Ea-b in S1 Supporting Information), the effect of eliminating emissions from all sectors at the same time yields much stronger benefits. This non-linear effect is consistent with results from Carslaw, Lee [34] on the effect of anthropogenic emissions on cloud albedo. Removing air pollutants from only one sector will leave large irradiance gains unutilized. It is thus important to focus efforts on all sectors, not only from a health perspective as any removal of air pollutants contributes to improve health [2].

**Fig 5:**
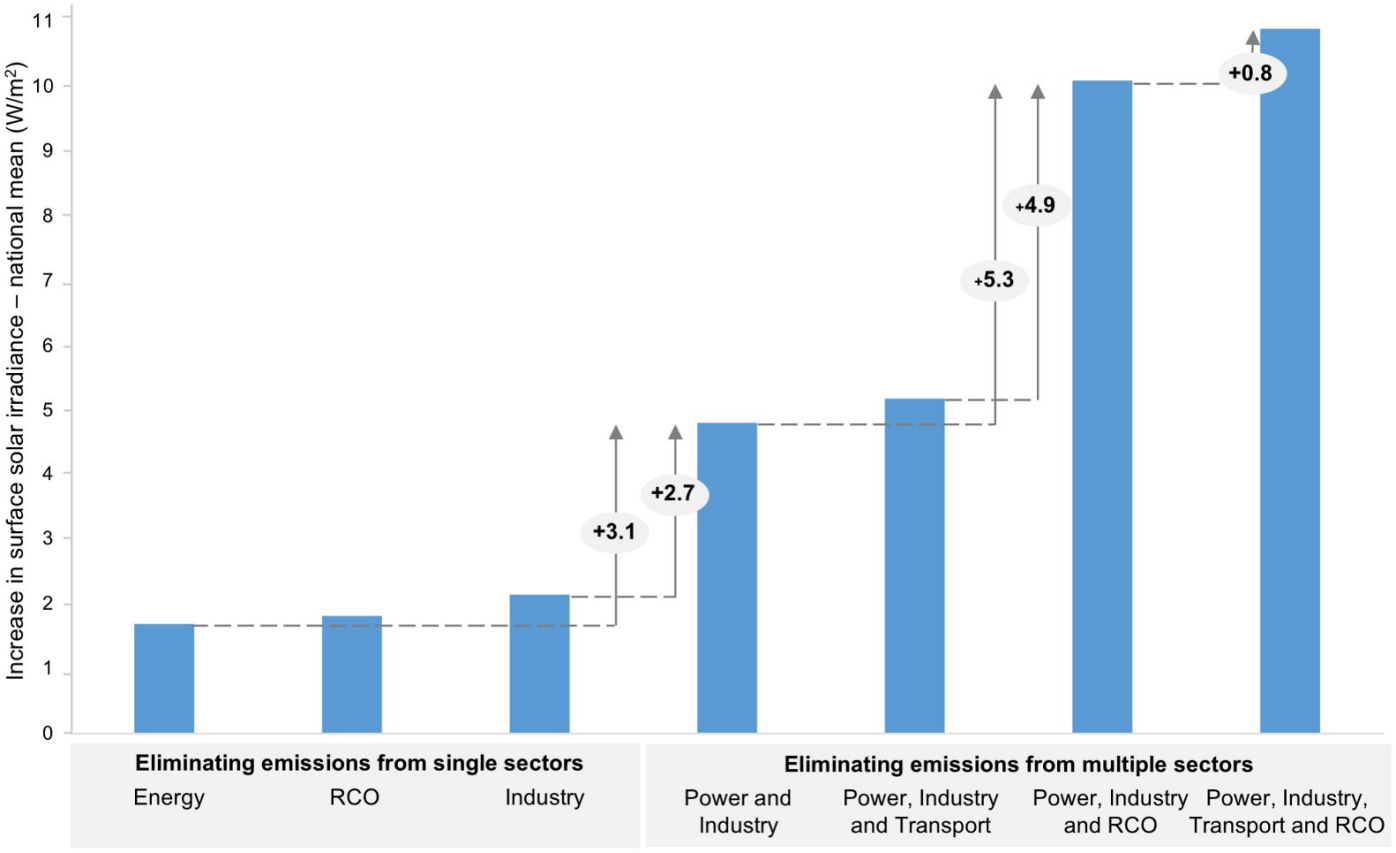
Breakdown of increases in surface solar irradiance (national mean, W/m^2^). From an elimination of SO_2_, BC, and OC emissions from single sectors: the energy, residential and commercial (RCO) or industrial sectors; and from multiple sectors at the same time: the energy and industrial sectors, both sectors with RCO, and all four sectors together. The national mean is the area-weighted mean increase in surface solar irradiance. The numbers are valid if pollution control happens for the stated single or combined strategies, as the effect is not linear: the relative contribution of each sector is sensitive to the order of pollution measures. Data and material from [19, 24–26].

Fig 5 summarizes the effect of adopting a multi-sector approach to eliminate all emissions compared to eliminating emissions from single sectors only on the average national surface solar irradiance. Haze pollution became a primary concern for air quality in most Chinese cities, especially those in the Beijing-Tianjin-Hebei region. As a result, local governments in this region aim to change the heating systems in the residential sector from coal to natural gas burning because of the significant contribution of residential emissions to local air pollution [40]. Here we show that the effect of eliminating residential emissions only, or those from the energy and industry sectors only on surface solar irradiance is smaller than when adopting a multi-sector sector approach. The largest effect on surface solar irradiance will be achieved when not only the emissions from the energy and the industrial sectors, i.e., the sectors that have received the most attention regarding reducing their emissions, are eliminated completely but also the emissions from the RCO sector. Once emissions from the RCO sector are removed, surface solar irradiance increases by more than twice as much as with eliminating emissions from the energy and industrial sectors only, as well as by more than five times as much with eliminating emissions from the energy sector only.

### Effect of clean-air policies on surface solar irradiance: Province-specific

**Fig 6:**
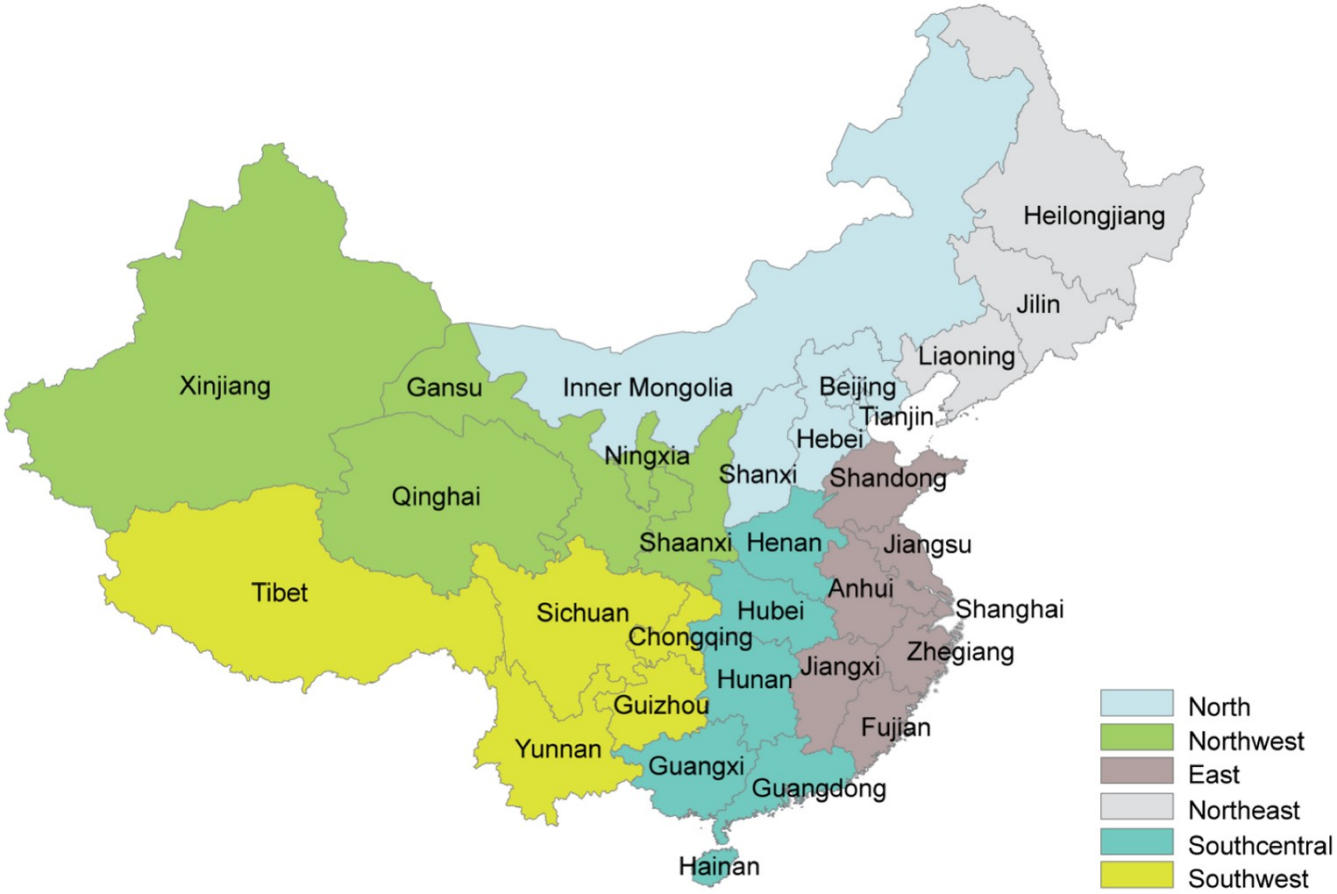
Map of the 31 provinces and the 6 regions of China.

**Fig 7:**
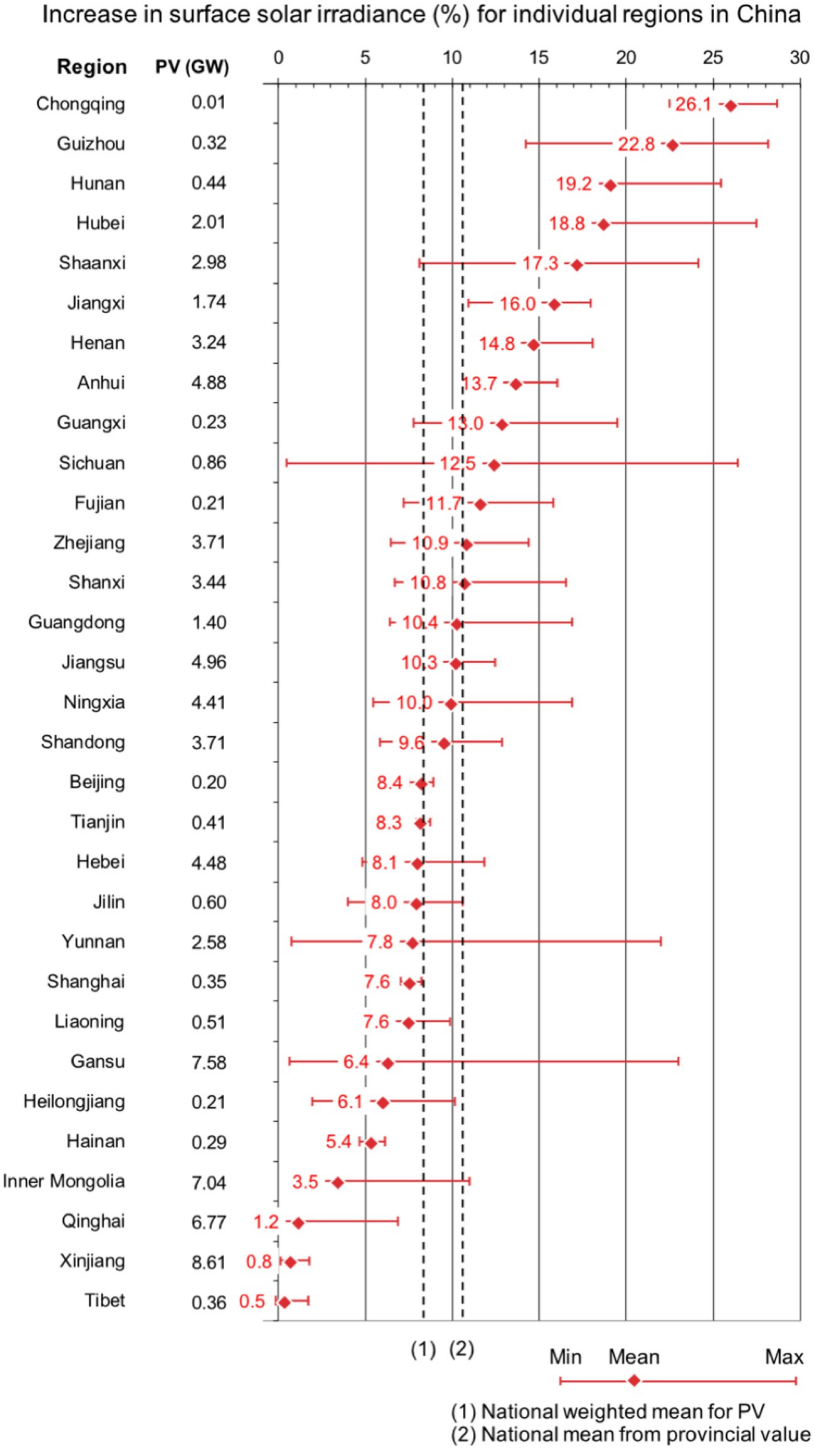
Increase in surface solar irradiance (min, max, mean %). For eliminating actual SO_2_, BC and OC emissions from all sectors. The mean province-specific increases in surface solar irradiance are area-weighted means. The national weighted mean for PV (8.3%) is weighted for province-specific PV capacities. The national mean from provincial values (10.6%) is the unweighted mean of all provinces. Right to the regions is the solar PV installed capacity (GW) per province as of December 2016. Data and material from [19, 24–26].

As seen in Figs 4 and 5 above, the effects of pollution control on irradiance can be strong, but they are geographically heterogeneous. Disaggregating the effect to province-specific numbers reveals just how much this can be. The provinces located in the geographic center of China (Chongqing, Guizhou, Hunan, Hubei, Shaanxi, Jiangxi, Henan), where the irradiance effect is strongest, could increase irradiance by 15%-28%, but because these regions have only ~10% of total installed PV capacity, the economic impact on the national PV fleet would be small. One-third of the Chinese solar PV capacity is installed in areas in Inner Mongolia, Qinghai, Xinjiang and Tibet far away from both demand and pollution centers: there, eliminating emissions would increase surface solar irradiance by less than 5%. Also, in these regions, the effect of pollution on the PV fleet is small. See Fig 6 for the location of the provinces, Fig 7 for province-specific increases in solar surface irradiance for eliminating emissions from all sectors, and Table J in S1 Supporting Information for detailed results for an elimination of actual emissions from the energy sector only, from both the energy and industrial sectors, with the transport or the RCO sectors, and from all sectors.

Most of the Chinese PV fleet (60%), however, is located near the population centers in the northern, eastern, and southcentral regions, where emissions are high, reducing surface solar irradiance by 5–15%. Given the large current and expected PV capacity of not only large-scale PV plants [11] but also decentralized PV [41]–as it is beneficial to build PV on rooftops due to the absence of transmission costs, stringent air pollution control measures in these regions will clearly increase the solar power output and hence the profitability of Chinese solar power.

### Revenues in the solar industry

The elimination of all aerosol species from all sectors would have increased the generation of the 2016 Chinese solar PV fleet by 10 TWh, or some 14% of the current solar PV generation [42]. Today’s solar PV generation in China represents about 1% of the final electricity consumption [42], thus the increase in electricity generation compared to the final electricity consumption is still minor. For future projected solar PV fleets, the effect would be 24 TWh for 2020 and 49–73 TWh for 2030, considering a low and a high scenario regarding installed solar PV capacity (Tables J and K in S1 Supporting Information for specific increases in solar generation). The increased Chinese PV generation in 2030 is roughly equal to the current electricity demand of a mid-sized European country, such as Austria [43]–just from the cleaner air, without a single MW of additional PV installation. By 2040, depending on the installed PV capacity, solar generation could increase to about 85–158 TWh.

The increase in surface solar irradiance would increase the revenues of the solar industry from the FiTs, as the generation of a given solar PV capacity increases: in 2016, removing all actual aerosols emissions from all sectors would have created US$1.4 billion in additional revenue from increased generation in the clearer air. This amount is equal to the economic losses from the curtailment of solar power as a result of the grid instability experienced in the same year [44]. By 2040, the revenues from increased solar PV generation could reach up to US$6.9 billion/year for a discount rate of 5% and when the FiTs decrease over time, and up to US$10.1 billion/year for the same discount rate but for FiTs the same as in 2017. See Table L in S1 Supporting Information for specific revenues for 2020, 2030, and 2040, for different discount rates, learning rates and capacity expansion scenarios; and Table M for specific non-discounted revenues.

### Cost-benefit ratios

The cost of measures in all sectors to reach near-zero emissions amounts to US$48.6–68.6 billion, while the revenues depend on several factors as described above, and in 2040 they could reach up to US$10.1 billion/year, see Tables N and O in S1 Supporting Information for numerical NPV results.

**Fig 8:**
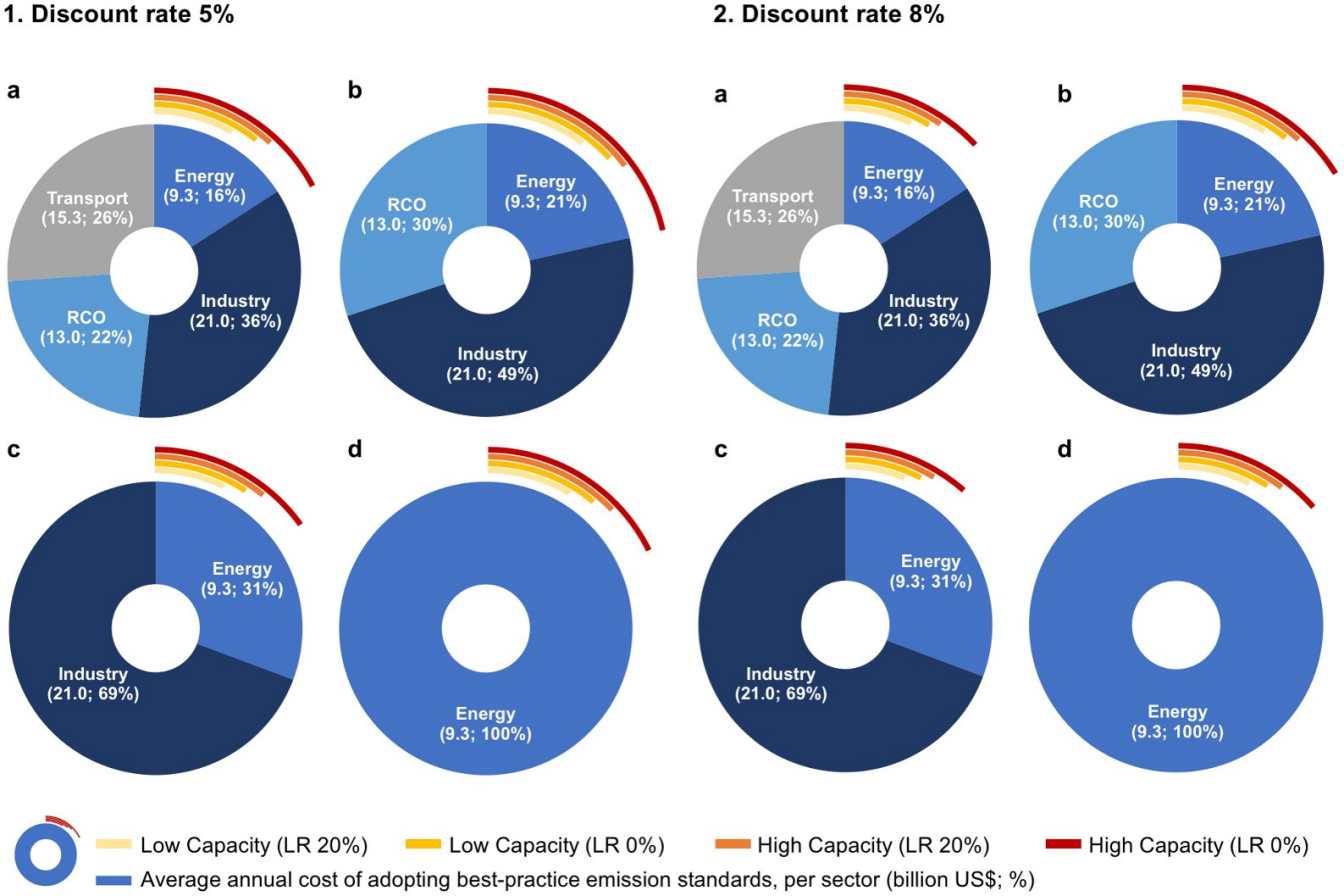
8-1 and 8-2 Annual average cost (billion US$ and %) of adopting best-practice emission standards. To all sectors (a), the energy, industrial and residential and commercial (RCO) sectors (b), the energy and industrial sectors (c), and the energy sector alone (d), compared to the annual revenues (billion US$, discounted) leveraged from the feed-in tariff on the Chinese PV fleet in 2040 for a low (700 GW) and a high capacity scenario (1300 GW), and for a feed-in tariff that reduces over time as the national PV system cost reduces following a technological learning rate of 20% starting in 2017, i.e., the year of the last available feed-in tariffs, and for a feed-in tariff without technological learning, i.e., equal to the feed-in tariffs in 2017. Revenues discounted to the present using a discount rate of 5% and 8%. Sector-specific annual costs are averages of a low and a high cost scenario, for a break-down of sub-sector-specific costs and uncertainty ranges see Table I in S1 Supporting Information. Data and material from [19, 24–26].

The cost compensation is highly dependent on the size of the PV fleet and FiTs, which are dependent on the technological learning rate: the scenarios with technological learning result in lower profit margins over time as compared to the scenarios without technological learning. Thus, the increased revenues in 2040 could compensate for about 13–17% of the pollution control cost in all sectors and about 16–21% of the combined pollution control cost in the energy, industrial, and RCO sectors (Fig 8 1a-b and Fig 8 2a-b; Figs G and H in S1 Supporting Information for results for 2020 and 2030), revealing a low level of marginal cost compensation for eliminating pollutants in the transport sector. Some 14–18% of the cost of eliminating emissions from the energy sector only—which given the past progress could be the sector where strong pollution control could be fastest to implement, could be offset via increased PV revenues. In this case, the revenues and the costs would arise in the same sector—electricity generation—potentially allowing for a direct link between the two, especially if the same actors own both coal and PV generators.

## Conclusion

Cleaning the air in China is possible through an expansion of best-practice measures for pollution control already implemented in China and elsewhere. Although the cost of doing so would be substantial, it would be societally beneficial—the health impacts of air pollution in China are a two-digit share of GDP. Here, we have shown that the elimination of SO_2_ emissions and carbonaceous aerosols will make the air clearer and increase surface solar irradiance, thereby strongly increasing the generation of solar PV electricity. The additional revenue would amount to up to about 20% of the cost of the pollution control measures, showing that there are hard economic benefits for cleaner air as well, in addition to the softer and hard-to-monetize health benefits. Hence, the increased revenue from the relatively minor solar industry already goes a long way towards justifying radical air pollution measures.

We have shown that reducing air pollution is not only an important health and environmental policy, but can also be an important solar power policy measure. Already-implemented policies to decrease the levels of air pollution have aimed to reduce the negative impacts on health but, as a co-effect, have also increased surface solar irradiance and hence solar generation by up to 3.5%. However, the largest effects of past efforts can be seen in regions where the installed PV capacity was comparatively low. The elimination of emissions could increase PV generation from the PV fleet by on average 11%. For the projected Chinese PV fleet of 2030, this could amount to the current power demand of Austria, only from the clearer air and without investing in a single additional PV array. The current PV expansion strategy increases this effect: in 2014–2016, the PV capacity in northwestern China, where skies are still relatively clear, doubled—already a remarkable expansion pace—but in the eastern provinces, where many of the most polluted regions and thus the haziest skies are found, it increased four-fold. This emphasizes the need for pollution control: the highest emissions occur in places where the bulk of the PV fleet is located and where capacity increases fastest. This is also where most people live and where pollution control will have the largest impact on health.

The Chinese government has made improving air quality a priority on its agenda for the upcoming years. Our results suggest that there are large economic benefits of doing so, especially if pollution control occurs in all sectors and all pollutants are included. As the magnitude of surface solar irradiance changes depends non-linearly on emission reductions, the stronger the emission reductions—multiple sectors together and stringent regulations in all—the larger the increase on solar power generation. The energy sector, which has seen the strongest emission policies in the past, may be the easiest to de-pollute, and it is directly affected by the revenue increases we identify here. Energy companies must bear the costs of pollution control in their power stations, and energy companies—potentially the same companies—will be the ones benefitting from the increased solar generation due to the cleaner air, providing the government with economic arguments for rapidly pushing ahead in the energy sector. Overall, urgent action is needed to clear the air in China, primarily because of the health impacts of air pollution, and we have shown that the side-effect of increased solar power generation would offset a sizeable share of the costs of air pollution control measures.

## Supporting information

S1 Supporting Information (PDF)
