# Supplementary material for: Blue skies over China: The effect of pollution-control on solar power generation and revenues
Source: PLoS One. 2018 Nov 21;13(11):e0207028. doi: 10.1371/journal.pone.0207028 (PMC6248963; doi:10.1371/journal.pone.0207028)
Supplement: S1 Supporting Information — (PDF) [file pone.0207028.s001.pdf]

Supporting Information

Blue skies over China: the effect of pollution-control on solar power generation and revenues

Mercè Labordena[1*], David Neubauer[2], Doris Folini[3], Anthony Patt[1], Johan Lilliestam[4]

[1] Climate Policy Research Group, Institute for Environmental Decisions, ETH Zurich, Zurich, Switzerland

[2] Atmospheric Physics Research Group, Institute for Atmospheric and Climate Science, ETH Zurich, Zurich, Switzerland

[3] Climate and Water Cycle Group, Institute for Atmospheric and Climate Science, ETH Zurich, Zurich, Switzerland

[4] Renewable Energy Policy Group, Institute for Environmental Decisions, ETH Zurich, Zurich, Switzerland

*Corresponding author

E-mail: merce.labordena@usys.ethz.ch (ML)

1 Cost of adopting best-practice emission standards in fossil-fueled sectors

1.1 Electricity generation for public use and plant consumption

Near-zero emissions power plants are equipped with a combination of multiple emission-removal technologies [1], consisting of three stages: the removal of particle matter (PM) with a fabric filter or with a dry electrostatic precipitator (ESP) equipped with a low-temperature economizer, as well as during desulfurization through a high-efficiency wet flue gas desulfurization (WFGD) system. The ESP or fabric filter has a PM removal efficiency of 99.8–99.9%, which reduces the concentration of PM to below 20 mg/m^3. Emissions of SO_2 are controlled using a high-efficiency WFGD system with a removal efficiency of ~99%, which also removes and additional ~50% of PM entering the system. This synergetic PM removal reduces the concentration of PM to below 15–10 mg/m^3. In a final step, a wet ESP removes and additional ~70% of PM, which reduces the concentration of PM to below 5 mg/m^3. A full-load denitration system, together with low-NO_x combustion in the boiler, has a NO_x-removal efficiency of ~85%, which reduces NO_x emissions to 40–10 mg/m^3.

The Shenhua Guohua Power Company has been an early adopter of near-zero emissions technologies. Retrofitting coal-fired power plants to reach near-zero emissions increases electricity generation costs. The incremental generating costs of reaching near-zero emissions, after accounting for the investments for retrofitting and being discounted over 15 years, for the plants Zhoushan No.4, Dingzhou No.3, and Sanhe No.1 are US$ 0.1, 0.1, and 0.16 ¢/kWh, respectively [2]. When comparing the total generating costs of a near-zero emissions plant to those of a natural gas combined-cycle plant, the generating costs of a natural gas combined-cycle unit in the province of Zhejiang is US 9.36 ¢/kWh, while those for Zhoushan No.4 in the same province is US 3.1 ¢/kWh – about one-third as much [2].

Retrofitting coal-fired power plants with the above-described combination of emission-removal technologies to reach near-zero emissions will increase electricity generation costs by US$ 0.1–0.16 ¢/kWh [2]. Because the thermal electricity generation (~90% coal-fired) in China in 2014 was 4,222 TWh [3], which accounts for the plants that generate electricity for sale to third parties and for the electricity used in plants for their own purposes, the cost of retrofitting the thermal plants is between US$4.2 and 6.7 billion/year.

We assume that the costs of retrofitting the combustion process for heat generation from electric boilers plants and the combustion processes in transformation processes with the same combination of air pollution control systems as is used for electricity generation are proportional to the level of SO_2 emissions and thus amount to US$1.5–2.3 billion/year and US$1.5–2.4 billion/year, respectively.

Hence, the total retrofitting cost in the energy sector, i.e., electricity and heat generation plants and transformation processes, amounts to US$7.2–11.4 billion/year.

1.2 Industrial combustion and industrial processes

Industrial boilers provide heat or process steam to meet the needs of the facilities in which they are installed. These facilities can be parts of the iron and steel industry, the chemical and petrochemical industry, the non-ferrous metals and non-metallic minerals industry, etc. While emissions from coal-fired power plants and coal-fired industrial boilers are affected by a number of variables such as coal type and composition and the type of combustion technology, the emission control technologies used to limit emissions from stack gases are essentially the same [4]. Hence, we assume that coal-fired industrial boilers are retrofitted with the same combination of air pollution control systems as is used in coal-fired power plants and that the cost is proportional to the level of SO_2 emissions and thus amount to US$12.0–19.1 billion/year.

It is also possible to replace the use of coal in industrial boilers with another, less-polluting fuel, such as natural gas. In many cases, converting coal-fired boilers to gas-fired boilers can be

profitable because the changes to the equipment are likely to be less expensive than installing air pollution control equipment; also, the use of natural gas would lead to lower emission characteristics.

China's natural gas production is rising at a fast pace but not fast enough to meet the demand required by the government to clean the country's air. China has the world's largest reserves of shale natural gas, and much of it could be recovered if cost were not a limitation [5]. The boilers and stoves used in the residential and commercial sectors are difficult to retrofit with effective pollution control equipment because of the small scale and age of the units, and it is also difficult to ensure that these units operate correctly. Hence, we assume that the residential and commercial sectors will switch to natural gas, while the industrial boilers will be equipped with the same pollution control equipment as is used in coal-fired power plants.

We discuss the industrial processes in the main text in the section *Clean-air policies and their cost*.

1.3 Road transport and domestic navigation

Gasoline and diesel fuels contain sulfur because it is a natural component of crude oil. The sulfur content in the fuel is the most important parameter affecting the introduction of measures to limit end-of-the-pipe emissions: A fuel with a sulfur content of ~50 ppm (parts per million) allows for the use of diesel particulate filters with an efficiency of ~50% and for the selective catalytic reduction (SCR) of NO_x with an efficiency of ~80%. In contrast, a fuel with near-zero sulfur content of ~10 ppm enables huge advances in fuel-efficient vehicle design and advanced control technology because it allows for the use of NO_x absorbers with an efficiency of over 90%, which enables engine designs with higher fuel efficiency and particulate filters that achieve an efficiency close to 100%, thereby emitting ~99% less $PM_{2.5}$ than uncontrolled vehicles [6].

The costs of reducing sulfur content in the fuel depend on the state of existing refineries, current fuel quality, and emissions standards but such costs can be divided into two types: the cost associated with fuel production and the cost associated with vehicle emission control technologies. Estimates of the costs associated with fuel production accounts for upfront refinery investment, such as capital equipment upgrades, and direct operating costs, such as catalysts and chemicals [7]. The costs of upgrading China's refineries to produce near-zero sulfur 10 ppm gasoline and diesel fuels are US 0.7¢ and US 1.7¢ per liter, respectively [8], which is comparable to international experiences and equivalent to 0.6–1.5% of the pump price. This translates into a total investment requirement of US$4.3 billion/year after accounting for upfront refinery investments, such as capital equipment upgrades, and direct operating costs, such as catalysts and chemicals [7].

Estimates of the cost for the introduction of advanced emission control technologies in vehicles account for the additional costs to manufacturers for equipping these vehicles with advanced emission control technologies to meet international best-practice standards, i.e., the adoption of the China 6 standard in gasoline and diesel vehicles. Table A shows the additional costs for manufacturers for equipping a vehicle with advanced emission control technologies to meet the China 6 standard, which for the current vehicle fleet amounts to US$7.4 billion/year [7].

Table A Additional cost (US$) per vehicle for manufacturers when they are compelled to install an emission control technology to meet the China 6 standard, over an uncontrolled emission level. Numbers adjusted based on engine size and labor and other expenses that are specific to China [7].

Fuel	Large Buses	Private Cars	Light Trucks	Heavy Trucks
	Diesel	Gasoline	Diesel	Diesel
China 6	4,765	366	4,248	9,200

Note: Costs reflect the incremental cost to manufacturers, not the price increment paid by the consumer. The additional costs are multiplied by the number of projected vehicles sold for each vehicle type by fuel type. The vehicle fleet does not account for motorcycles.

The adoption of international best-practice standards such as ultra-low sulfur standards and the China 6 standard, will lead to a total additional cost of US$11.7 billion/year. These costs assume that all vehicles that emit a disproportionate share of total emissions, known as “yellow-label vehicles,” will be scrapped. Although scrappage is a near-term measure to reduce air pollution, China is implementing one of the most ambitious voluntary scrappage programs for old vehicles worldwide because it will result in rapid urban air quality improvements.

China has introduced standards regarding the content of sulfur in the fuels used for navigation, to limit the content of sulfur to 3.5% m/m (mass of sulfur/total mass, or 35,000 ppm). As of 1 January 2018, vessels that operate in the Yangtze River Delta, the Pearl River Delta, and the Bohai Sea should use fuels with a sulfur content of less than 0.5% m/m. The International Maritime Organization (IMO) will extend this limit to all international ports two years later [9]. Ships navigating in sulfur emission control areas, i.e., the North Sea, the Baltic Sea, the English Channel, and the North American coasts, should use fuels with a sulfur content of less than 0.1% m/m. China is also considering reducing the allowable sulfur content to 0.1% m/m, which will reduce SO_x emissions by ~97%.

There is not a single way forward to reduce or eliminate sulfur and related emissions from navigation. The options include continuing to use heavy fuel oil (HFO) while also cleaning the exhaust gas with scrubbers; switching to a low-sulfur fuel, such as marine gas oil (MGO) or liquefied natural gas (LNG); or a combination of both. Thus, ship-owners will have to choose whether to invest in scrubbers or use low-sulfur fuel, a choice they will make based on the ship’s age, the price of scrubbers and their operational costs, and the price differential between high-sulfur and ultra-low-sulfur fuels.

Switching from HSFO to MGO or MDO with a sulfur content of 0.1% m/m is a straightforward solution for carriers because engines do not need to be retrofitted with emission control technologies to accept this type of fuel, although minor adjustments in auxiliary equipment are needed in some cases. The costs of equipping an average-size medium-range ship of 10MW to

adapt to MDO or MGO, i.e., installing a fuel cooler or chiller and the associated piping prior to the fuel pump to decrease fuel viscosity, and also SCR technology, are about US$0.8 million and US$0.5 million, respectively; and the operational cost derived from the use of the reducing agent in SCR is about US$0.2 million per year [10, 11]. The capital costs of SCR systems, which vary with engine design, are the stronger determinant of the system's costs. In general, the larger the engine, the less expensive the installation costs are per MW.

China has about 2,400 Chinese-owned oceangoing container vessels in its waterways [12], a number in line with the 2,444 vessels provided by the World Factbook, which are comprised of 1,069 bulk carriers, 198 container ships, 697 general cargos, and 480 oil tankers [13]. If 2,400 container vessels switch from HSFO to MGO at a cost of US$1.5 million per ship, the total cost of the container fleet switching fuels will be US$3.6 billion/year.

China has also about 10,500 coastal vessels, including small passenger ships, fishing boats, etc., and 147,200 river vessels [12]. Most of the fishing boats and small passenger ships already operate with low-sulfur fuel oil due to their limited sizes and hence limited engine capacities.

Overall, low oil prices favor solutions with the lowest capex, i.e., MGO, while high oil prices favor solutions with a higher capex, i.e., scrubbers or liquefied natural gas (LNG). Under stricter international emission standards, the demand for scrubbers may increase, and the costs may go down as production scales. Also, the price of HSFO is expected to fall sharply when the cap set by the IMO comes into force in 2020, while the price of ULSFs is expected to dramatically increase. Thus, the use of scrubbers may be the most cost-effective way for larger ships to comply with the sulfur limit.

Emissions from the transport sector originate not only from road transport and navigation, but also from the combustion processes in rail transport. Emissions from rail transport account for 3% of SO_2 emissions from the transport sector, or 1% of total SO_2 emissions from all sectors. Thus, because emissions from rail transport are not significant, we exclude rail transport when estimating the cost of reaching near-zero emissions. Hence, the total cost of reducing emissions in the transport sector due to road transport and domestic navigation is US$15.3 billion/year.

1.4 Residential and commercial sector

Households and businesses in China burned 119 million tons of coal in 2014, which accounted for 4% of total national consumption [3]. Both the boilers employed to heat residential and commercial buildings and stoves used for cooking lack effective pollution control equipment and retrofitting them with such equipment is difficult because of the age and small scale of the units, as well as the difficulty of ensuring that these units operate correctly. Also, the cost of retrofitting the units may be too high for poor owners.

To significantly improve air quality at the urban level, the burning of coal and other pollution sources, such as wood, biomass, and waste, can be replaced with natural gas or propane. Also, it is possible to switch to electrical appliances fed with electricity from low-emission sources, though the prior method is the most efficient way because the equipment is already in place. To reduce air pollution levels, cities and villages in Northern China and, more notoriously, the nation's capital, Beijing, have started to replace coal-fired residential heating and cooking with gas-powered stoves and boilers.

Table B shows that to replace coal in residential and commercial uses with natural gas or propane, China would need to procure an additional 93.86 billion m^3 of natural gas, which would represent a 70% increase over the total of 159.32 billion m^3 of natural gas consumed in 2014 [3]. In our calculations, we assume that the switch is made to a single fuel, i.e., natural gas, because in East Asia the costs per unit of energy of natural gas and propane are similar. We also assume that one energy unit of natural gas can substitute for one energy unit of coal in household and commercial users; which is a conservative assumption because the new natural gas heating devices are likely to be more efficient than old coal-fired boilers.

Table B Replacing coal with natural gas for residential and commercial use in China.

	Coal Use (Mtce)	Conversion	Natural gas equivalent (billion m^3)
**Residential rural**	78.11	0.786	61.39
**Residential urban**	14.42	0.786	11.33
**Commercial**	26.90	0.786	21.14
**Total**	**119.43**		**93.86**

During the first half of 2017, average natural gas pipeline import prices in China averaged US$187 per m^3, while LNG import prices averaged US$253 m^3 [14]. At a price of US$187 per m^3, the cost of an additional 93.86 billion m^3 of natural gas is US$17.55 billion, and at a price of US$253 per m^3, the cost is US$23.74 billion.

Table C shows the annual net cost of replacing coal with natural gas for residential and commercial use. The average prices of imported anthracite and imported bituminous coal are US$58.90 per ton and US$68.76 per ton, respectively [3]. Subtracting the average value of the coal for which these fuels would substitute, at an average coal price of US$63.83 per ton, this results in US$7.62 billion. Hence, the net annual costs are from US$9.9 billion to US$16.1 billion/year. We acknowledge the volatility of the price of natural gas, which can influence the results. The import prices for natural gas via pipeline and LNG during 2015-2016 were rather stable, but in previous years, the prices had been more than 50% higher.

Table C Annual cost of replacing coal with natural gas for residential and commercial use.

Policy measure	Quantity natural gas (billion m^3)	Price natural gas (US$ per m^3)	Total annual cost (billion US$)	Net annual cost (billion US$)
Price pipeline import	93.86	187	17.55	9.93
Price LNG import	93.86	253	23.74	16.12

We do not estimate the investment cost of converting a coal-fired boiler to a natural gas-fired boiler, because of the lack of available data on the capacity of installed residential and

commercial boilers and the cost of replacing those boilers. It is likely that the costs of converting boilers will represent a small share of the total costs and savings from shifting from coal to natural gas or propane. Also, switching from coal to gas involves the construction of natural gas distribution networks, pipelines, and household connection facilities, the prices of which are uncertain. Hence, we acknowledge these uncertainties and exclude these estimates from this analysis.

2 Evaluation of the global aerosol-climate model ECHAM6.3-HAM2.3

A comprehensive evaluation of the individual factors contributing to the modelled surface solar irradiance in ECHAM6.3-HAM2.3 including cloud cover and aerosol indirect effects is in preparation and will be published elsewhere. Here, we answer the question of how realistic modelled surface solar irradiance under all-sky conditions, i.e., including clouds, is in China. We also examine the plausibility of modelled surface solar irradiance changes under changing anthropogenic aerosol emissions.

Regarding the first question, we show that ECHAM6.3-HAM2.3 captures well the overall magnitude and spatial pattern of all-sky surface solar irradiance in China. Fig A compares the annual mean surface solar irradiance from ECHAM6.3-HAM2.3 (remapped onto the CERES grid and CEDS aerosol emission data for the year 2014 [15]) and CERES (from 2005 to 2015 [16]) for China and neighboring regions. Fig Aa-b show an overall similar range of surface solar irradiance for ECHAM6.3-HAM2.3 and CERES of ~100-250 W/m^2 and a similar geographical distribution: particularly low surface solar irradiance in parts of eastern and south-central China (notably around 30N and 105E, in the basin area of Chengdu and Chongqing), as well as particularly high surface solar irradiance in parts of western China and the Tibetan plateau with its high elevation. Fig Ac shows that the difference in surface solar irradiance

between the two data sets is within ±10 W/m^2 in most parts of China, particularly in eastern China, where the majority of the population resides.

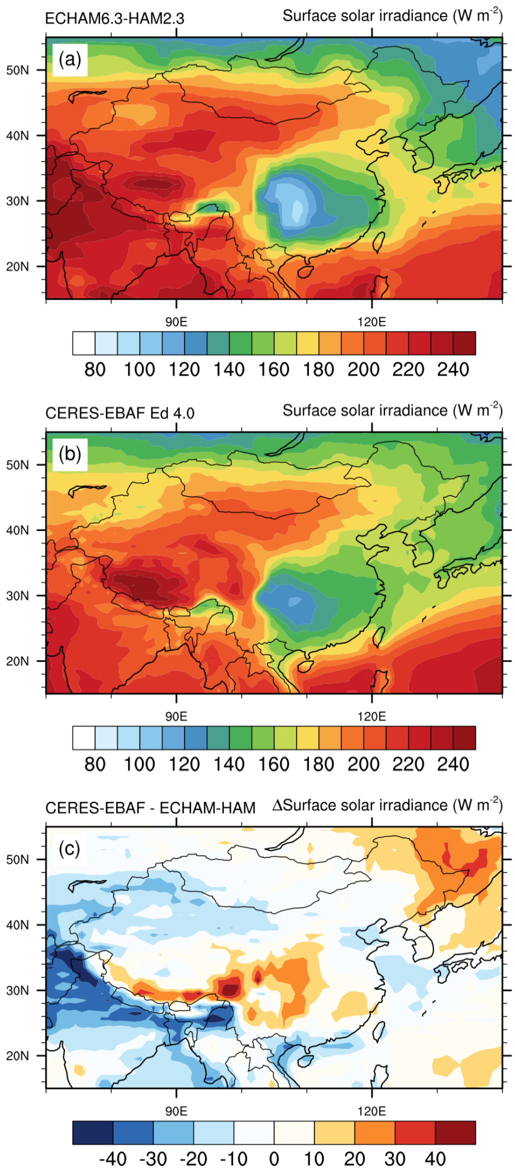

Fig A Annual mean surface solar irradiance (W/m^2). Annual mean surface solar irradiance from ECHAM6.3-HAM2.3 (a) [17-19], CERES-EBAF (b) [16], and the difference of CERES-EBAF and ECHAM6.3-HAM2.3 (c); created with [20].

The largest differences (dark red) are found around 30N and 100E, a region of very steep topography as the terrain ascends towards the Himalaya / Tibetan Plateau and where, consequently, neither the modeled nor the satellite data with their comparatively coarse spatial resolution can be considered very reliable.

Similar considerations apply to the reddish patches further to the west, in the flanks of the Himalaya, as well as to the dark blue patches in the north west, the Tian Shan mountains. In the remaining reddish and bluish areas, differences between ECHAM6.3-HAM2.3 and CERES are mostly smaller than ±20 W/m^2. Reasons for these differences comprise again topography, but also natural and anthropogenic aerosols as well as clouds and aerosol-cloud interactions and water vapor.

In the west, the overestimation of surface solar irradiance in ECHAM6.3-HAM2.3 with respect to CERES (bluish colors) is equally present for clear sky conditions, which indicates that aerosols or water vapor play a role for the difference. Yet water vapor as the only cause seems questionable given that the difference is between 10-20 W/m^2. A reason can be an underestimation of natural aerosol, notably mineral dust, in ECHAM6.3-HAM2.3. An underestimation of anthropogenic aerosol emission seems less likely, as such emissions are comparatively small in this region in the first place. The latter is in line with the fact that there is essentially no observed change in surface observations of sunshine duration since 1960 (Figure 5 in [21]). The difference between modeled and satellite data is dependent also on the contribution from clouds / cloud properties, and particularly in the central region of China clouds show a strong seasonality (monsoon).

In the reddish area from 100E to 110E and from 26N to 32N, where the surface solar irradiance from ECHAM6.3-HAM2.3 is 30-40 W/m^2 lower than in CERES, the situation is slightly more complicated as this is a hot spot region of anthropogenic aerosol emissions, as given in CEDS emissions data. Again, the difference seen in all sky surface solar irradiance is in line with the clear sky difference. This again indicates that aerosols play a role for the difference between modeled and satellite, but the influence of clouds cannot be excluded.

After comparing the results from ECHAM6.3-HAM2.3 and CERES satellite data, we note that studies comparing surface solar irradiance from CERES with surface solar irradiance from surface observations find a general overestimation of surface solar irradiance in China by CERES on the order of 10W/m^2, see [22] Figure 4 and Table 6 and [23] Figure 3 and Table 5, which confirm the results of ECHAM6.3-HAM2.3 regarding the range and spatial pattern of all-sky surface solar irradiance in China.

Regarding the second question, we show that modeled surface solar irradiance changes are in line with observation-based estimates of surface solar irradiance reduction from anthropogenic aerosol emissions since the 1960s. We have shown that in eastern China, changes in modeled solar irradiance are up to ~25 W/m^2 and in the central region up to ~30 W/m^2 (Fig D and Table J). Published estimates of anthropogenic aerosol induced dimming in China show a total reduction from the 1960s till today of 20 W/m^2 or more (Figure 5 in [24]; Figure 3c in [25]; Figure 10 in [26]; Figure 5 in [21]). These studies show that the observed reduction of surface solar irradiance is strongest in the eastern and central parts of China and it is similar to our results; yet these studies also show diverse results as to when and where in eastern China the observed reduction of surface solar irradiance is the strongest. A more detailed comparison of modeled surface solar irradiance in ECHAM6.3-HAM2.3 with observation-based estimates is beyond the scope of this study.

In summary, absolute all-sky surface solar irradiance is modeled reasonably well by ECHAM6.3-HAM2.3 and that modeled, anthropogenic aerosol induced surface solar irradiance changes are plausible as compared to published observation-based estimates of changes in solar irradiance.

3 Policies to control and reduce air pollution

China has been implementing policies to control air pollution for more than three decades. However, it was not until the Ninth Five-Year Plan (FYP) (1996-2000) that the first target to

limit total national SO_2 emissions was introduced. The target was few million tons higher than actual emissions, and it was achieved. The Tenth FYP (2000-2005) set a national target to reduce SO_2 emissions by 10% by 2005 as compared to 2000 levels. Policies to control emissions included equipping new and existing coal-fired power plants with desulfurization systems; the phase-out of small, inefficient power plants with poor technology and high pollution discharges; the replacement of these power plants with larger, more efficient units; a higher pollution levy rate; and stricter emission standards. At the end of the period, about 14% of the coal-fired capacity had desulfurization systems installed, yet less than half of these were running continuously and reliably [27, 28]. These policies proved insufficient to meet the national target.

The Eleventh FYP (2006-2010) held the same national emissions target, but it was stricter in some provinces and implemented more ambitious policies [29]. These policies included a doubling of the pollution levy rate, stricter regulations on the closure of small, less-efficient power plants, a price premium for electricity generated from plants with desulfurization systems, financial assistance for the installation of desulfurization systems, and fines to plants if such systems were in operation less than 90% of the time. As a result of these measures, SO_2 emissions from the electric power plants began to fall in 2006, with a reduction of about 36% from 2006 to 2010 (Fig 2 in main text). The percentage of coal-fired capacity with desulfurization systems increased to 86% in 2010 [30]. This time, the policies proved sufficient to meet the national target.

The Twelfth FYP (2011-2015) established air pollution prevention and control measures not only in the power sector but also in high-emitting industries, including the iron and steel, cement, pulp and paper, non-ferrous metals, and flat glass industries, as well as on transport. The plan introduced, for the first time, politically binding targets for SO_2, NO_x, and CO_2 intensity [31]. The emission standards became stricter and were comparable to those in Europe and the United States. The Emission Standard for Air Pollutants from Thermal Power Plants limited SO_2 emission concentrations for new and existing coal-fired plants to 100 mg/m^3 and

200 mg/m^3, respectively, except in some provinces where the coal used to fuel the plant has a high sulfur content. There, higher emission limits were allowed [31]. For *key regions* of pollution control, which account for more than 66% of China's GDP, the limit was 50 mg/m^3.

In 2013, a winter-long episode of severe haze over many provinces and cites in eastern China became worldwide news. The concentration of $PM_{2.5}$ in Beijing, for instance, was 40 times the limit recommended by the World Health Organization [32]. As a consequence, air pollution control policies were strengthened on multiple fronts. The China National Action Plan on Air Pollution Prevention and Control (2013–2017), the country's toughest move to reduce air pollution, set limits to coal consumption for the power and industrial sectors. For the industrial sector, the plan also included the promotion of technology upgrades, stricter controls on high-polluting and energy-intensive industries, and the shutdown of the most polluting factories [33]. The plan also set stricter air quality standards for the most polluted eastern regions, i.e., the Beijing-Tianjin-Hebei, Yangtze River Delta, and Pearl River Delta regions.

In 2014, the Coal Energy-Saving Upgrade and Transformation Action Plan (2014–2020) went into force, requiring all new and existing plants to limit SO_2 emissions to 35 mg/m^3 by 2020 [34]. Shenhua's Sanhe Power Plant 1, one of the plants on which we based the cost estimates, was China's first coal-fueled unit that successfully underwent a retrofit to comply with the emissions limit [1].

4 Counterfactual scenarios

The counterfactual scenarios represent the emissions of the energy sector if no pollution control policies had been introduced since 2006 and the emissions of the industrial sector if the same pollution control policies as used for the energy sector had been applied to industry as well. We estimate emissions in the counterfactual scenarios for SO_2, BC, and OC, we do so in two steps.

First, we estimate the emissions factor for the energy sector for the year before the policies to control air pollutants from coal-fired power plants became effective. We calculate the emissions

factor as the emission estimates divided by the energy consumption at a regional level for the 22 provinces, 4 municipalities and 5 autonomous regions for 2005; see Equation (A1). We use province-level data on *Fuel use for power generation* as a driver of emissions from the energy sector [3]. Because the Chinese National Bureau of Statistics does not report energy consumption data for Tibet but it does report thermal power generation, we use the last as a driver for the energy sector. We convert thermal power generation (TWh) into the units given for fuel use (million tonnes of coal equivalent, Mtce), multiplying TWh by the yearly average heat rates for fossil fuel-fired power plants depending on their average fuel consumption [3]. This assumption does not significantly influence the results, because thermal power generation in Tibet is very low.

$$\boldsymbol{EF_{power\,(i)}} = \frac{\boldsymbol{E'_{power\,(i,2005)}}}{\boldsymbol{EC_{power(i,2005)}}} \quad \text{(A1)}$$

Second, we estimate the emissions for the *Energy counterfactual* scenario by multiplying the emission factor at the province-level by the province-level *Fuel use for power generation* ($Mtce_{2006\text{-}2014}$); see Equation (A2).

$$\boldsymbol{E_{power\,(i,j)}} = \boldsymbol{EC_{power\,(i,j)}}\ \boldsymbol{EF_{power\,(i)}} \quad \text{(A2)}$$

where $E'_{power\,(I,\,2005)}$ is the actual emissions (Mt) in province i for year 2005, $EC_{power\ (I,\,2005)}$ is the fuel use for power generation (Mtce) in province i for 2005, $E_{power\,(I,\,j)}$ is the emissions (Mt) at province (i) for year (j), $EC_{power\ (I,\,j)}$ is the fuel use for power generation (Mtce) at province (i) for year j, and $EF_{power\,(i)}$ is the emission factor (Mt/Mtce) in province i for 2005.

We calculate the emissions for the *Industry counterfactual* scenario as described in Equation (A3). We use province-level data on *Industrial sector end use* [3] as a driver of emissions from industry. The data on *Industrial sector end use* are given as coal, petroleum, and electricity consumption. We convert the coal and petroleum consumption given in Mt into Mtce, using the same procedure as described for the energy sector.

$$\boldsymbol{E_{industry(i,j)}} = \frac{\boldsymbol{E'_{industry\,(i,j)}}}{\frac{\boldsymbol{E_{power\,(i,j)}}}{\boldsymbol{E'_{power\,(i,j)}}}} \quad \text{(A3)}$$

where $E_{industry\ (I,\ j)}$ is the emissions (Mt) in province i for year j, $E'_{industry}$ is the actual emissions (Mt) in province i for year j, $E_{power\ (I,j)}$ is from Equation (A2), and $E'_{power\ (I,j)}$ is the actual emissions (Mt) in province i for year j.

5 Pollutants by sector

Table D Pollutants from the energy, industrial, residential and commercial, and transport sectors in China for 2014. Reactive gas: sulfur dioxide (SO_2), and carbonaceous aerosols: black carbon (BC) and organic carbon (OC). Units: Kilotonnes (kt) [15].

**Sectors**	**Pollutants, kt**		
**Energy**	**SO_2**	**BC**	**OC**
**Combustions emissions**			
Electricity public and auto-producer	5,596	63	139
Heat production	1,956	7	16
Transformation (e.g., fuel combustion in coal coke production, oil refining, charcoal production)	1,998	712	1,187
**Non-combustions emissions**			
Fugitive petroleum and gas	0	3	1
Fossil-fuel fires	199	0	0
**Sub-total:**	**9,749**	**785**	**1,343**
**Industry**			
**Combustions emissions**			
Iron and steel	3,143	38	29
Non-ferrous metals	371	5	4
Chemicals	2,369	30	22
Pulp and paper	543	7	5
Food and tobacco	822	11	8
Non-metallic minerals	6,781	83	61
Construction	21	14	5
Machinery	361	8	4
Mining and quarrying	227	10	4
Other	656	7	4
Textile leather	419	6	4
Transport equipment	166	4	2
Wood products	119	2	1
**Non-combustions emissions**			
Chemical industry	262	0	0
Metal production	5,090	0	0
Pulp and paper. Food and beverage. Wood	304	0	0

**Sub-total:**	**21,654**	**225**	**152**
**Residential and Commercial**			
**Combustions emissions**			
Commercial-institutional	925	95	108
Residential	3,226	823	1,810
Agriculture, forestry and fishing	762	79	83
Other	771	54	70
**Sub-total:**	**5,684**	**1,051**	**2,071**
**Transport**			
**Combustions emissions**			
Road	41	206	86
Rail	8	16	4
Domestic navigation	179	12	9
Other	7	2	1
**Sub-total:**	**235**	**237**	**100**
**Total:**	**37,322**	**2,299**	**3,666**

6 Installed solar PV capacities

Table E Historical cumulative installed solar PV capacities (MW) by province and region [35].

Region	Province	2010	21011	2012	2013	2014	2015	2016
**East**	Anhui	7	7	42	209	580	1'745	4'876
	Fujian	6	11	37	59	114	168	208
	Jiangsu	90	388	492	1'524	2'586	3'763	4'930
	Jiangxi	10	16	65	116	155	442	1'737
	Shandong	59	176	259	530	819	2'020	3'732
	Shanghai	19	16	24	193	228	319	348
	Zhejiang	10	31	111	370	828	1'674	3'704
	**Subtotal:**	**200**	**645**	**1'030**	**3'001**	**5'310**	**10'132**	**19'535**
**North**	Beijing	4	22	73	130	195	197	197
	Hebei	6	55	99	489	1'083	2'454	4'423
	Inner Mongolia	13	128	335	1'371	3'380	5'293	7'023
	Shanxi	10	34	49	227	563	1'231	3'387
	Tianjin	5	19	21	99	134	191	412
	**Subtotal:**	**38**	**258**	**576**	**2'316**	**5'354**	**9'366**	**15'441**
**NorthEast**	Heilongjiang	-	30	56	68	78	148	209
	Jilin	2	2	2	72	77	180	643
	Liaoning	0	33	46	94	114	185	505
	**Subtotal:**	**2**	**65**	**104**	**234**	**268**	**513**	**1'357**
**NorthWest**	Gansu	16	253	960	4'487	6'358	7'141	7'561
	Ningxia	96	410	593	1'493	2'223	3'549	4'360
	Qinghai	65	901	1'427	3'710	5'222	6'086	6'768
	Shaanxi	9	38	87	248	811	1'478	2'977
	Xinjiang	-	102	453	3'307	4'169	6'529	8'515
	**Subtotal:**	**186**	**1'704**	**3'520**	**13'244**	**18'784**	**24'784**	**30'181**
**SouthCentral**	Guangdong	20	32	104	279	523	893	1'396
	Guangxi	-	1	1	21	30	160	231
	Hainan	-	20	54	137	207	277	289
	Henan	3	15	52	127	523	813	3'236
	Hubei	9	16	30	96	138	1'153	2'008
	Hunan	5	38	60	138	256	373	491
	**Subtotal:**	**37**	**122**	**300**	**798**	**1'677**	**3'670**	**7'651**
**SouthWest**	Chongqing	-	-	-	-	-	-	10
	Guizhou	-	-	-	-	-	50	320
	Sichuan	11	11	14	76	126	456	903
	Tibet	10	90	154	208	228	288	361
	Yunnan	92	178	220	340	555	1'854	2'581
	**Subtotal:**	**114**	**279**	**388**	**624**	**909**	**2'648**	**4'174**
	**Total:**	**577**	**3'073**	**5'919**	**20'217**	**32'302**	**51'112**	**78'341**

7 Correction factors on solar PV generation

Table F Correction factors on the annual electricity generation solar for optimal panel orientation and tilt for each province. Values obtained from CM SAF SARAH solar radiation data set [36]. Some provinces fall out of the range of the satellites; hence, we assign to the provinces marked with (*) the correction factors of the neighbor Anhui and with (**) of the neighbor Beijing.

Province	Correction factor	Province	Correction factor
**East**		**Nord-West**	
Anhui	1.072	Gansu	1.196
Fujian	1.044	Ningxia	1.187
Jiangsu	1.089	Qinghai	1.146
Jiangxi	1.032	Shaanxi	1.126
Shandong	1.122	Xinjiang	1.197
Shanghai*	1.072	**South-Central**	
Zhejiang*	1.072	Guangdong	1.048
**Nord**		Guangxi	1.029
Beijing	1.174	Hainan	1.014
Hebei	1.154	Henan	1.086
Inner Mongolia	1.219	Hubei	1.053
Shanxi	1.162	Hunan	1.032
Tianjin	1.154	**Nord-West**	
**Nord-East**		Chongqing	1.026
Heilongjiang**	1.174	Guizhou	1.026
Jilin**	1.174	Sichuan	1.037
Liaoning**	1.174	Tibet	1.163
		Yunnan	1.084

8 Feed-in tariff schemes

Table G shows the various feed-in tariffs (FiTs) schemes for on-grid solar photovoltaic (PV) projects in China over the years. As specified by the *Renewable Energy Law of the People's Republic of China*, since 2009, there has been an indemnificatory purchasing system in place for solar power generation. To determine the acceptable level of FiT, project tenders were invited in two rounds: the first in 2009, for a 20 MW project in the province of Gansu; the second in 2010, for a group of projects totaling 280 MW in Shaanxi, Qinghai, Inner Mongolia, and Ningxia. In 2011, the National Development and Reform Commission (NDRC) issued the *Notice on Perfecting a Feed-in Tariff Policy of Solar Energy PV Power Generation*, which determined the benchmark for the first nationwide, unified FiT. That FiT did not account for the differences in solar radiation across China. Hence, solar projects' owners focused on the installation of solar PV plants in western China, where energy demand is lower due to lower population density and economic development. To address this mismatch, in 2013, the NDRC issued the *Notice on Promoting the Healthy Development of the Solar PV Industry through the Price Leverage Effect,* dividing the FiT into three different compensation levels depending on solar resources and construction costs [37]; see Table H. The FiT was further reduced for those projects registered after the beginning of 2016.

Table G Feed-in tariffs for solar PV projects (RMB /kWh, including tax), rates over the years [38] [39] [40] [37] [41].

2009(1)	2010(2)	2011(3)	2012(3)	2013	Resource area	2014(4)	2016(5)	2017(6)
1.09	1.15; 0.73-0.99	1.15	1.00	1.00	Area I	0.90	0.8	0.65
					Area II	0.95	0.88	0.75
					Area III	1.00	0.98	0.85

[1] The feed-in tariff (FiT) for selected project applies since March 2009.

[2] The FiT for the projects in Ningxia was RMB1.15 /kWh, in April 2010. The FiT for the projects in Shaanxi, Qinghai, Inner Mongolia and Ningxia was RMB0.73 /kWh for the lowest, while RMB 0.99 /kWh for the highest, in June 2010.

[3] Nationwide FiT of RMB1.15 /KWh for projects completed and put into operation prior to December 31, 2011, and of RMB1.0 /KWh after that day in every province except Tibet, which enjoys the right to employ the former FiT.

[4] The FiT apply to projects registered after September 1, 2013. Projects that were registered before that date, but started generation after January 1, 2014, were also eligible for the subsidies. Tibet employ the FiT of RMB1.0 /KWh.

[5] The FiT apply to projects registered after January 1, 2016. Tibet employ the FiT of RMB1.0 /KWh.

[6] The FiT apply to projects registered after January 1, 2017. Tibet employ the FiT of RMB1.0 /KWh.

Table H Chinese regions within each the three resource areas [37].

Resource area	Regions within resource area
Area I	Ningxia (Ningxia Autonomous Region); Haixi (Qinghai Province); Jiayuguan, Wuwei, Zhangye, Jiuquan, Dunhuang, Jinchang (Gansu Province); Hami, Tacheng, Altay, Karamay (Xinjiang Autonomous Region); areas in Inner Mongolia other than Chifeng, Tongliao, Xing'anmeng and Hulunbeier.
Area II	Beijing (Beijing Municipality); Tianjin (Tianjin Municipality); Heilongjiang (Heilongjiang Province); Jilin (Jilin Province); Liaoning (Liaoning Province); Sichuan (Sichuan Province); Yunnan (Yunnan Province); Chifeng, Tongliao, Xing'anmeng and Hulunbeier (Inner Mongolia Autonomous Region); Chengde, Zhangjiakou, Tangshan and Qinhuangdao (Hebei Province); Datong, Shuozhou and Xinzhou (Shanxi Province); Yulin and Yan'an (Shaanxi Province), areas in Qinghai, Gansu and Xinjiang other than Resource Area I.
Area III	Areas other than areas in Resource Areas I-II, including Tibet autonomous region

Note: Regional solar resources as classified in class I, II or III in descending order of solar endowment.

9 Cost of implementing clean-air policies

Table I Annual costs (billion US$) of implementing policies in a sectorial basis to reach near-zero emissions.

Sector and sub-sectors		Low estimate	High estimate
**Energy**			
	**Combustion processes**		
	Electricity generation	4.2	6.7
	Heat generation	1.5	2.3
	**Non-combustion processes**		
	Transformation	1.5	2.4
	**Sub-total**	**7.2**	**11.4**
**Industry**			
	**Combustion processes**	12.0	19.1
	**Non-combustion processes**	4.2	6.7
	**Sub-total**	**16.2**	**25.8**
**RCO**			
	**Combustion processes**		
	Residential rural	6.5	10.6
	Residential urban	1.2	1.9
	Commercial	2.2	3.6
	**Sub-total**	**9.9**	**16.1**
**Transport**			
	**Combustion processes**		
	Road transport (fuel)	4.3	
	Road transport (engine)	7.4	
	Domestic navigation	3.6	
	**Sub-total**	**15.3**	
**Total**		**48.6**	**68.6**

10 Effect of counterfactual and potential clean-air policies on surface solar irradiance: nationwide

10.1 Effect of past and counterfactual clean-air policies on surface solar irradiance

Here we show the effect of past and counterfactual air pollution control measures on surface solar irradiance. In the energy sector, China has made strong progress, and visibility is better than it would have been without past and existing measures. Fig Ba shows the effect of the SO_2 control measures and the removal of carbonaceous particles in the energy sector since 2006, i.e., moving from *Energy counterfactual* to *Energy actual*, which have increased surface solar irradiance by up to 3.5% (5 W/m^2). Applying the same emission standards to industry as already exist for energy, i.e., reducing emissions from *Industry actual* to *Industry counterfactual*, would increase surface solar irradiance by up to 2.2% (1.2 W/m^2). If we assume that state-of-the-art pollution control with full coverage is applied in the energy sector, and that this “near-zero” emission technology will fully eliminate all aerosol emissions (i.e., from *energy actual* to zero), the irradiance would increase by up to 6% (10 W/m^2), showing that although much has been achieved in the energy sector, there is still much room for further improvement. Overall, the effect of pollution control on solar irradiance is large: eliminating all energy and industry emissions from unabated levels (*energy counterfactual* and *industry actual*), assuming no control measures since 2006, increases irradiance by up to 13.5% (16 W/m^2; Fig Ca-d for results expressed in W/m^2 irradiance increase).

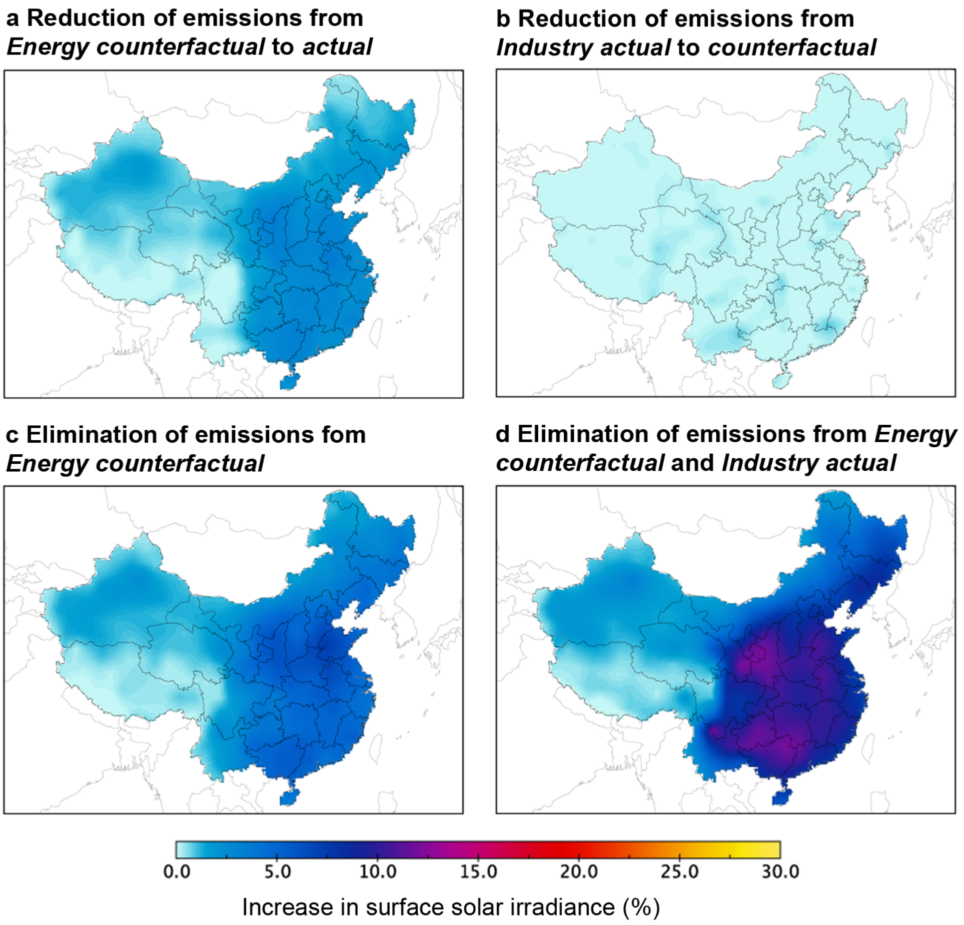

Fig B Increase in surface solar irradiance in percent (%). From an emission abatement of SO_2, BC, and OC emission from energy counterfactual to energy actual (a), from industry actual to industry counterfactual (b), from an elimination of emissions from energy counterfactual (c), and an elimination of emissions from energy counterfactual and industry actual (d). Data and material from [15, 17-19].

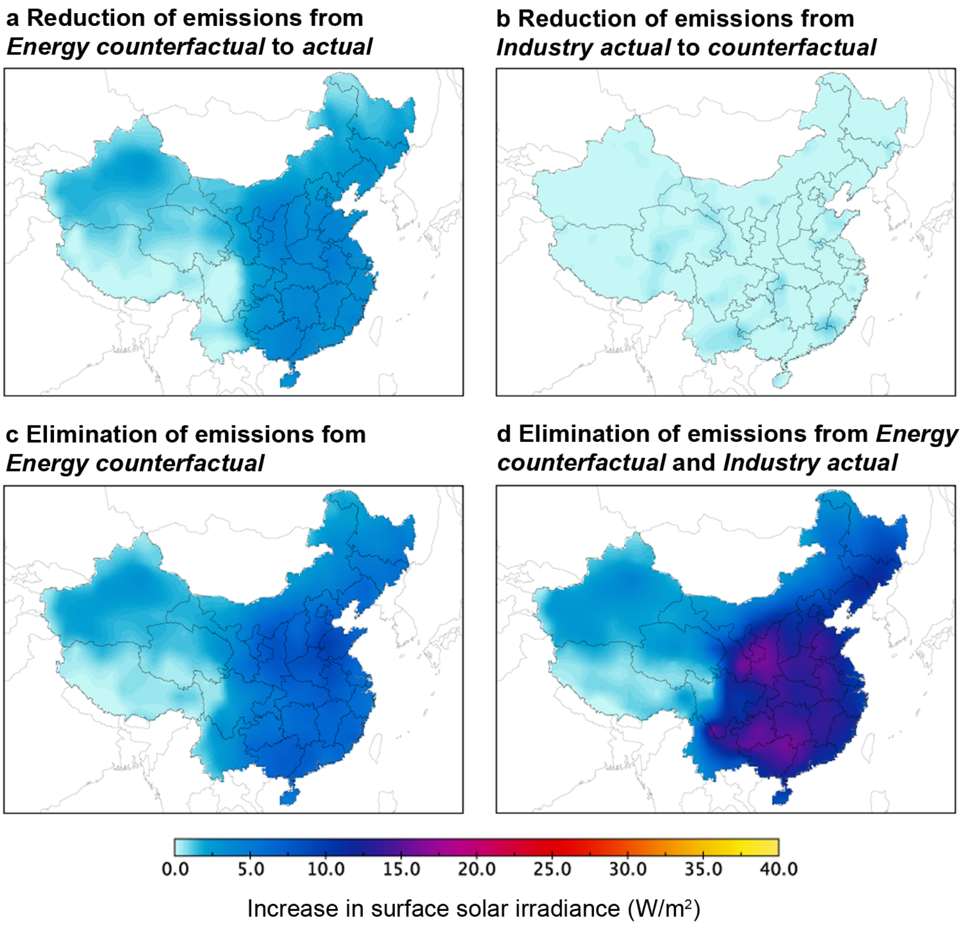

Fig C Increase in surface solar irradiance (W/m^2). From an emission abatement of BC, OC and SO_2 from energy counterfactual to energy actual (a), from industry actual to industry counterfactual (b), from an elimination of emissions from energy counterfactual (c), and an elimination of emissions both from energy counterfactual and industry actual (d). Data and material from [15, 17-19].

10.2 Effect of potential clean-air policies on surface solar irradiance

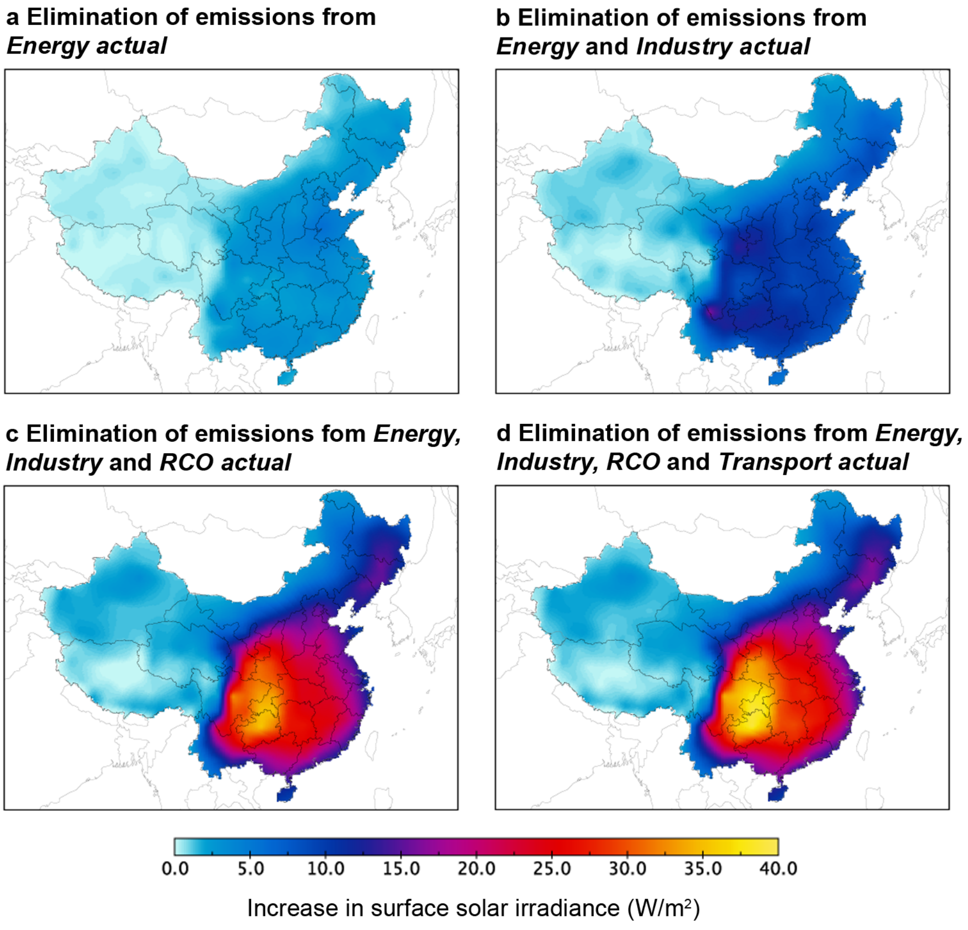

Fig D Increase in surface solar irradiance (W/m^2). From an elimination of actual BC, OC and SO_2 emissions from energy sector (a), of actual emissions from energy and industrial sectors (b), of actual emissions from energy, industrial and residential and commercial (RCO) sectors (c), and of actual emissions from energy, industrial, RCO, and transport sectors (d). Data and material from [15, 17-19].

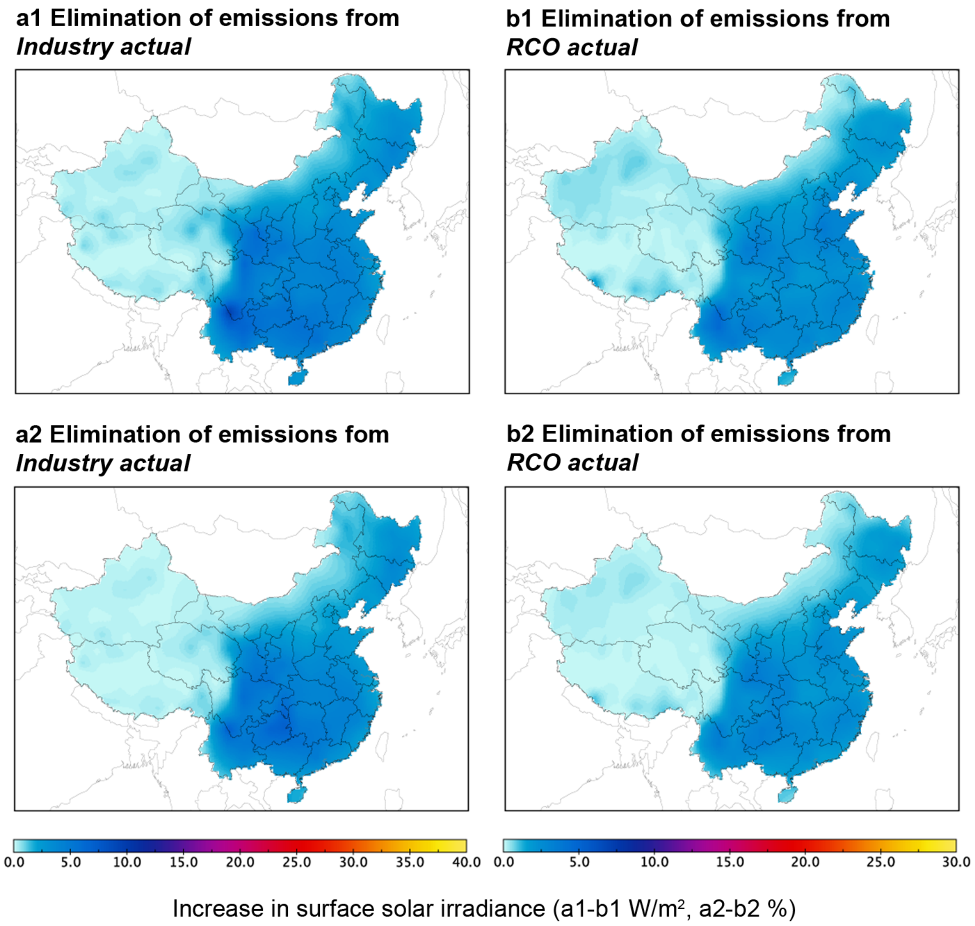

Fig E Increase in surface solar irradiance (W/m^2, %). From an elimination of actual BC, OC and SO_2 emissions from industrial sector (a), and an elimination of actual emissions from residential and commercial (RCO) sector (b). Data and material from [15, 17-19].

10.3 Effect of potential clean-air policies for SO_2 on surface solar irradiance

The solar radiation benefits of eliminating all current, actual emissions will be greater than if the focus were on eliminating SO_2 emissions only: eliminating all emissions in the energy sector increases surface radiation by up to 6 W/m^2, as compared to the increase of 2.4 W/m^2 seen when eliminating only SO_2 (Fig Fa-d). The irradiation gains from eliminating all emissions in the energy and industrial sectors is 16.8 W/m^2, as compared to the 14.4 W/m^2 from eliminating only SO_2. Also, the irradiation gains from eliminating all emissions in the energy, industrial and RCO sectors is 35.6 W/m^2, as compared to the 21.5 W/m^2 from eliminating SO_2.

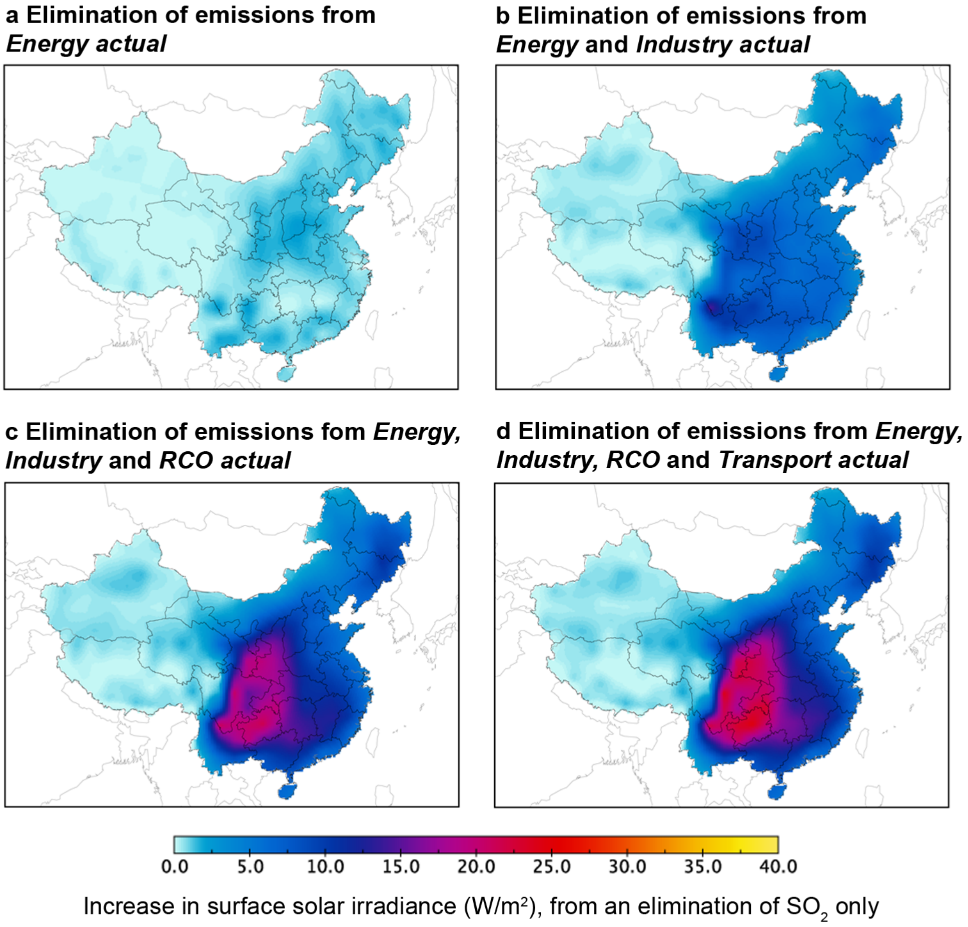

Fig F Increase in surface solar irradiance (W/m^2). From an elimination of actual SO_2 emissions from energy sector (a), of actual emissions from energy and industrial sectors (b), of actual emissions from energy, industrial and

residential and commercial (RCO) sectors (c), and of actual emissions from energy, industrial, RCO, and transport sectors (d). Data and material from [15, 17-19].

The solar radiation benefits of eliminating all aerosol emissions in the energy sector compared to eliminating in the same sector only SO_2 emissions (3.6 W/m^2) are greater than for the energy and industrial sectors combined (2.4 W/m^2). This non-linear increase in surface solar radiation is due to the relative amount of SO_2 emissions per sector compared to total emissions. As seen in Table D, the ratio of SO_2 emissions to total emissions in the energy and industrial sectors combined (0.98) is larger than in the energy sector alone (0.82). Thus, for the energy and industrial sectors combined, eliminating only SO_2 emissions is closer to eliminating all aerosol emissions than for the energy sector alone. Also, there is no one-to-one correspondence between the ratio of "SO_2 emissions to all emissions" and of "SO_2 induced irradiance reductions to irradiance reductions from all emissions" because the model computes effects of, e.g., aerosol mixing, aerosol cloud interactions, and different transport and deposition properties.

11 Province-specific increases in surface solar irradiance

Table J Mean province-specific increases in solar surface irradiance (W/m^2) and its corresponding increases in solar electricity generation (GWh per year) for the operational grid-connected solar PV installations as of December 2016, for an elimination of actual SO_2, BC and OC emissions from a number of emission reduction strategies on different combinations of sectors. The means are area-weighted means.

			Increase in solar surface irradiance			Increase in solar electricity generation
**Province**	**Energy**	**Energy and Industry**	**Energy, Industry and Transport**	**Energy, Industry and RCO**	**All sectors**	**All sectors**
Anhui	3.7	9.2	10.4	20.4	23.0	897.5
Beijing	3.2	8.0	8.7	15.1	16.2	28.2
Chongqing	3.2	10.0	10.8	33.4	37.0	2.8
Fujian	3.2	8.5	9.2	17.8	19.4	31.4
Gansu	3.3	5.7	5.9	11.0	11.6	786.0
Guangdong	2.9	8.9	9.5	17.7	18.7	203.9
Guangxi	2.9	10.0	10.6	20.7	22.0	38.9
Guizhou	2.4	11.6	12.3	31.0	33.7	82.4
Hainan	2.6	5.8	6.2	10.7	11.5	25.1
Hebei	1.7	7.7	8.7	14.4	15.6	600.8
Heilongjiang	3.1	5.0	5.3	9.1	9.4	17.1
Henan	2.7	9.8	10.9	22.7	25.7	671.4
Hubei	1.3	9.7	11.0	26.1	29.3	462.1
Hunan	2.2	10.3	11.5	26.5	29.2	97.9
Inner Mongolia	2.5	3.8	8.7	16.0	6.7	426.3
Jiangsu	1.6	8.0	10.9	23.3	17.8	713.6
Jiangxi	3.0	9.7	7.5	12.5	25.6	341.8
Jilin	0.8	7.2	7.8	12.9	13.3	70.3
Liaoning	3.4	7.6	4.1	6.3	13.6	60.3
Ningxia	1.2	9.7	10.0	18.7	19.7	768.8
Qinghai	3.1	1.5	1.7	2.4	2.5	146.0
Shaanxi	3.3	10.8	11.5	26.2	28.8	718.5

Shandong	2.8	8.8	9.9	16.5	18.0	557.4
Shanghai	3.5	6.4	6.7	11.6	13.0	36.6
Shanxi	3.2	8.7	9.4	18.4	20.2	602.2
Sichuan	3.3	7.4	7.7	18.4	19.9	132.9
Tianjin	2.7	8.3	9.2	15.1	16.0	56.5
Tibet	2.6	0.6	0.9	1.6	0.9	2.8
Xinjiang	0.7	1.6	0.6	0.8	1.7	128.7
Yunnan	0.3	7.7	7.9	14.3	14.8	308.7
Zhejiang	0.5	7.6	8.1	16.0	17.5	519.9
**Country-wide**	**1.7**	**4.8**	**5.2**	**10.1**	**10.9**	**9'537**

12 Increase in solar generation

Table K Increase in solar generation (TWh) from an elimination of emissions from a specific sector or a combination of them, for projected installed PV capacities for 2020, 2030 and 2040, the last two for a low and a high PV capacity scenario.

	Elimination of emissions			
	**Energy, Industry, RCO and transport**	**Energy, Industry and RCO**	**Energy and Industry**	**Energy**
**Low capacity scenario**				
**2020, 200 GW**	24.3	22.3	11.0	3.9
**2030, 400 GW**	48.6	44.6	22.0	7.8
**2040, 700 GW**	85.0	78.0	38.5	13.6
**High capacity scenario**				
**2020, 200 GW**	24.3	22.3	11.0	3.9
**2030, 600 GW**	72.9	66.9	33.0	11.7
**2040, 1300 GW**	157.9	144.9	71.6	25.3

13 Total revenues from increase in solar generation

Table L Total annual revenues (billion US$, discounted) leveraged from the feed-in tariff on the increase in solar power, for feed-in tariffs that reduce over time as the national PV system cost reduces following a technological learning rate of 20% starting in 2017, i.e., the year of the last available feed-in tariffs, for feed-in tariffs without technological learning, i.e., equal to the feed-in tariffs in 2017, and for projected installed PV capacities for 2020, 2030 and 2040, the last two for a low and a high PV capacity scenario. Revenues discounted to the present using a discount rate of 5% and 8 % (*).

Increased annual revenues in the solar sector from elimination of sectoral emissions				
	**Energy, Industry, RCO and transport**	**Energy, Industry and RCO**	**Energy and Industry**	**Energy**
**Learning rate 0%**				
**Low PV capacity scenario**				
**2020, 200 GW**	2.9 – 2.8*	2.6 – 2.6*	1.3 – 1.3*	0.5 – 0.5*
**2030, 400 GW**	4.6 – 4.1*	4.2 – 3.8*	2.1 – 1.9*	0.8 – 0.7*
**2040, 700 GW**	6.2 – 5.1*	5.7 – 4.7*	2.8 – 2.3*	1.0 – 0.8*
**High PV capacity scenario**				
**2020, 200 GW**	2.9 – 2.8*	2.6 – 2.6*	1.3 – 1.3*	0.5 – 0.5*
**2030, 600 GW**	6.4 – 5.5*	5.9 – 5.0*	2.9 – 2.5*	1.1 – 0.9*
**2040, 1300 GW**	10.1 – 7.7*	9.3 – 7.1*	4.6 – 3.5*	1.7 – 1.3*
	**Energy, Industry, RCO and transport**	**Energy, Industry and RCO**	**Energy and Industry**	**Energy**
**Learning rate 20%**				
**Low PV capacity scenario**				
**2020, 200 GW**	2.7 – 2.6*	2.5 – 2.4*	1.2 – 1.2*	0.5 – 0.5*
**2030, 400 GW**	3.9 – 3.6*	3.6 – 3.3*	1.8 – 1.6*	0.7 – 0.6*
**2040, 700 GW**	4.9 – 4.1*	4.4 – 3.8*	2.2 – 1.9*	0.8 – 0.7*
**High PV capacity scenario**				
**2020, 200 GW**	2.7 – 2.6*	2.5 – 2.4*	1.2 – 1.2*	0.5 – 0.5*
**2030, 600 GW**	5.1 – 4.5*	4.7 – 4.1*	2.3 – 2.0*	0.8 – 0.8*
**2040, 1300 GW**	6.9 – 5.6*	6.4 – 5.1*	3.3 – 2.6*	1.2 – 1.0*

Table M Total annual revenues (billion US$, undiscounted) leveraged from the feed-in tariff on the increase in solar power, for feed-in tariffs that reduce over time as the national PV system cost reduces following a technological learning rate of 20% starting in 2017, i.e., the year of the last available feed-in tariffs, for feed-in tariffs without technological learning, i.e., equal to the feed-in tariffs in 2017, and for projected installed PV capacities for 2020, 2030 and 2040, the last two for a low and a high PV capacity scenario.

Increased annual revenues in the solar sector from elimination of sectoral emissions				
	**Energy, Industry, RCO and transport**	**Energy, Industry and RCO**	**Energy and Industry**	**Energy**
**Learning rate 0%**				
**Low PV capacity scenario**				
**2020, 200 GW**	3.1	2.8	1.4	0.5
**2030, 400 GW**	5.8	5.3	2.6	1.0
**2040, 700 GW**	9.9	9.1	4.5	1.6
**High PV capacity scenario**				
**2020, 200 GW**	3.1	2.8	1.4	0.5
**2030, 600 GW**	8.6	7.9	3.9	1.4
**2040, 1300 GW**	18.2	16.7	8.2	2.9
	**Energy, Industry, RCO and transport**	**Energy, Industry and RCO**	**Energy and Industry**	**Energy**
**Learning rate 20%**				
**Low PV capacity scenario**				
**2020, 200 GW**	2.9	2.7	1.3	0.5
**2030, 400 GW**	4.7	4.3	2.1	0.8
**2040, 700 GW**	7.1	6.5	3.2	1.2
**High PV capacity scenario**				
**2020, 200 GW**	2.9	2.7	1.3	0.5
**2030, 600 GW**	6.5	6.0	3.0	1.1
**2040, 1300 GW**	11.2	10.3	5.5	2.0

Table N Net present value (NPV) (billion US$) for costs of policies to reach near-zero emissions (low estimate) and for discounted revenues. Revenues discounted to the present using a discount rate of 5% and 8 % (*). A positive value means a net cost of the sectoral policy.

NPV of sectoral emission elimination policies				
	**Energy, Industry, RCO and transport**	**Energy, Industry and RCO**	**Energy and Industry**	**Energy**
**Learning rate 0%**				
**Low PV capacity scenario**				
**2020, 200 GW**	45.7 – 45.8*	30.8 – 30.9*	22.1 – 22.1*	6.7 – 6.7*
**2030, 400 GW**	44.0 – 44.5*	29.7 – 30.0*	21.3 – 21.5*	6.4 – 6.5*
**2040, 700 GW**	42.4 – 43.5*	28.9 – 29.5*	20.6 – 21.1*	6.2 – 6.4*
**High PV capacity scenario**				
**2020, 200 GW**	45.7 – 45.8*	30.8 – 30.9*	22.1 – 22.1*	6.7 – 6.7*
**2030, 600 GW**	42.2 – 43.1*	28.6 – 29.2*	20.5 – 20.9*	6.1 – 6.3*
**2040, 1300 GW**	38.5 – 40.9*	26.9 – 28.2*	18.8 – 19.9*	5.5 – 5.9*
	**Energy, Industry, RCO and transport**	**Energy, Industry and RCO**	**Energy and Industry**	**Energy**
**Learning rate 20%**				
**Low PV capacity scenario**				
**2020, 200 GW**	45.9 – 46.0*	30.8 – 30.9*	22.2 – 22.2*	6.7 – 6.7*
**2030, 400 GW**	44.7 – 45.0*	29.7 – 30.0*	21.6 – 21.8*	6.5 – 6.6*
**2040, 700 GW**	43.7 – 44.5*	28.9 – 29.5*	21.1 – 21.5*	6.4 – 6.5*
**High PV capacity scenario**				
**2020, 200 GW**	45.9 – 46.0*	30.8 – 30.9*	22.2 – 22.2*	6.7 – 6.7*
**2030, 600 GW**	43.5 – 44.1*	28.6 – 29.2*	21.1 – 21.4*	6.4 – 6.4*
**2040, 1300 GW**	41.7 – 43.0*	26.9 – 28.2*	20.1 – 20.8*	6.0 – 6.2*

Table O Net present value (NPV) (billion US$) for costs of policies to reach near-zero emissions (high estimate) and for discounted revenues. Revenues discounted to the present using a discount rate of 5% and 8 % (*). A positive value means a net cost of the sectoral policy.

NPV of sectoral emission elimination policies				
	**Energy, Industry, RCO and transport**	**Energy, Industry and RCO**	**Energy and Industry**	**Energy**
**Learning rate 0%**				
**Low PV capacity scenario**				
**2020, 200 GW**	65.7 – 65.8*	50.7 – 50.7*	35.9 – 35.9*	10.9 – 10.9*
**2030, 400 GW**	64.0 – 64.5*	49.1 – 49.5*	35.1 – 35.3*	10.6 – 10.7*
**2040, 700 GW**	62.4 – 63.5*	47.6 – 48.6*	34.4 – 34.9*	10.4 – 10.6*
**High PV capacity scenario**				
**2020, 200 GW**	65.7 – 65.8*	50.7 – 50.7*	35.9 – 35.9*	10.9 – 10.9*
**2030, 600 GW**	62.2 – 63.1*	47.4 – 48.3*	34.3 – 34.7*	10.3 – 10.5*
**2040, 1300 GW**	58.5 – 60.9*	44.0 – 46.2*	32.6 – 33.7*	9.7 – 10.1*
	**Energy, Industry, RCO and transport**	**Energy, Industry and RCO**	**Energy and Industry**	**Energy**
**Learning rate 20%**				
**Low PV capacity scenario**				
**2020, 200 GW**	65.9 – 66.0*	50.8 – 50.9*	36.0 – 36.0*	10.9 – 10.9*
**2030, 400 GW**	64.7 – 65.0*	49.7 – 50.0*	35.4 – 35.6*	10.7 – 10.8*
**2040, 700 GW**	63.7 – 64.5*	48.9 – 49.5*	35.0 – 35.3*	10.6 – 10.7*
**High PV capacity scenario**				
**2020, 200 GW**	65.9 – 66.0*	50.8 – 50.9*	36.0 – 36.0*	10.9 – 10.9*
**2030, 600 GW**	63.5 – 64.1*	48.6 – 49.2*	34.9 – 35.2*	10.6 – 10.6*
**2040, 1300 GW**	61.7 – 63.0*	46.9 – 48.2*	33.9 – 34.6*	10.2 – 10.4*

14 Additional results

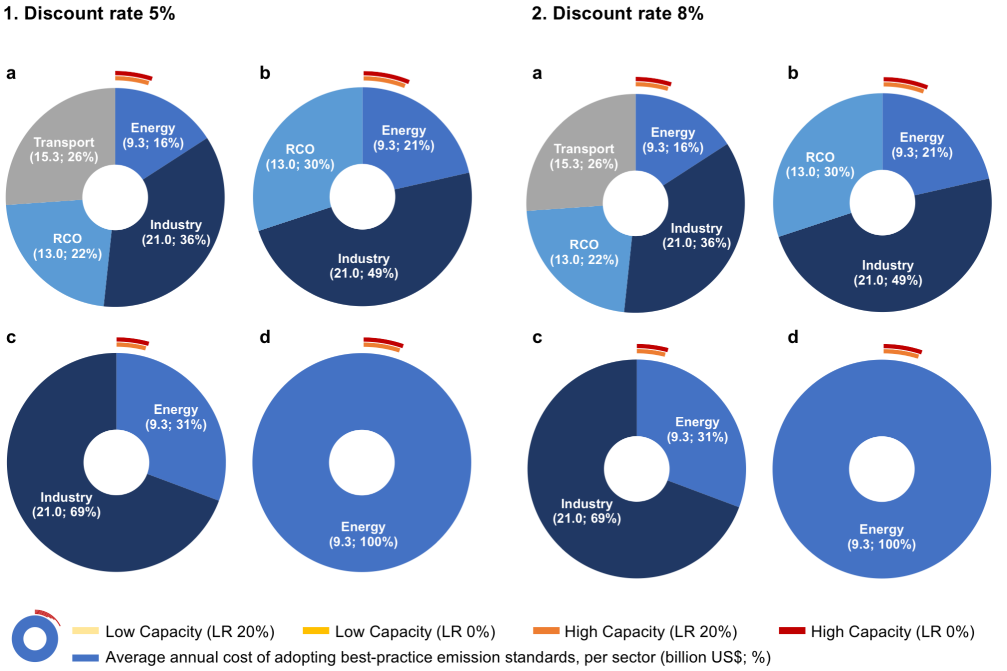

Fig G-1 and G-2 Annual average cost (billion US$ and %) of adopting best-practice emission standards. To all sectors (a), the energy, industrial and residential and commercial (RCO) sectors (b), the energy and industrial sectors (c), and the energy sector alone (d), compared to the annual revenues (billion US$, discounted) leveraged from the feed-in tariff on the Chinese PV fleet in 2020 of 200 GW, for a feed-in tariff that reduces over time as the national PV system cost reduces following a technological learning rate of 20% starting in 2017, i.e., the year of the last available feed-in tariffs, and for a feed-in tariff without technological learning, i.e., equal to the feed-in tariffs in 2017. Revenues discounted to the present using a discount rate of 5% and 8%. Sector-specific annual costs are averages of a low and a high cost scenario, for a break-d own of sub-sector-specific costs and uncertainty ranges see Table I. Data and material from [15, 17-19].

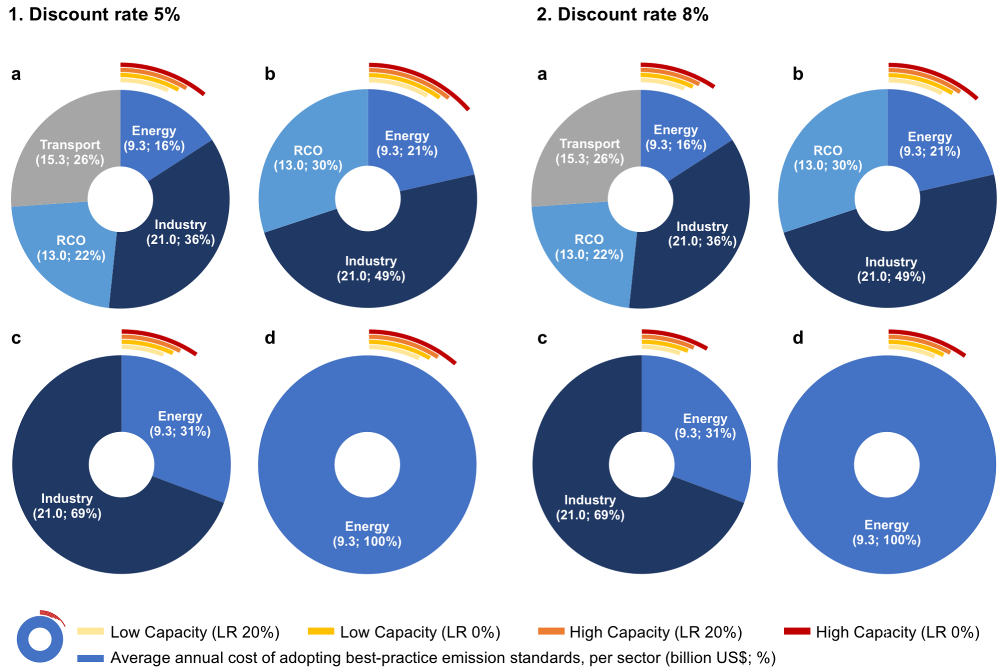

Fig H-1 and H-2 Annual average cost (billion US$ and %) of adopting best-practice emission standards. To all sectors (a), the energy, industrial and residential and commercial (RCO) sectors (b), the energy and industrial sectors (c), and the energy sector alone (d), compared to the annual revenues (billion US$, discounted) leveraged from the feed-in tariff on the Chinese PV fleet in 2030 for a low (400 GW) and a high capacity scenario (600 GW), and for a feed-in tariff that reduces over time as the national PV system cost reduces following a technological learning rate of 20% starting in 2017, i.e., the year of the last available feed-in tariffs, and for a feed-in tariff without technological learning, i.e., equal to the feed-in tariffs in 2017. Revenues discounted to the present using a discount rate of 5% and 8%. Sector-specific annual costs are averages of a low and a high cost scenario, for a break-down of sub-sector-specific costs and uncertainty ranges see Table I. Data and material from [15, 17-19].

References

1. World Coal Association. Case Study. Near-zero non-CO2 emissions. Shenhua Group. World Coal Association, 2017.

2. Shumin W. Shenhua Guohua’s Application of Near-Zero Emissions Technologies for Coal-Fired Power Plants: Cornerstone, 2015 [2018.02.26]. Available from: http://cornerstonemag.net/shenhua-guohuas-application-of-near-zero-emissions-technologies-for-coal-fired-power-plants/.

3. Fridley D, Lu H, Khanna N, Xu A, Zhu A. China Energy Databook v9.0. In: Laboratory CEGLBN, editor. Berkeley, CA, 2016.

4. World Bank. Environmental, Health, and Safety Guidelines: International Finance Corporation (IFC) World Bank Group, 2018 [2018.02.15]. Available from: http://www.ifc.org/wps/wcm/connect/topics_ext_content/ifc_external_corporate_site/sustainability-at-ifc/policies-standards/ehs-guidelines.

5. South China Morning Post. China has massive reserves, so why can’t it meet demand for gas? South China Morning Post, 2018 [2018.03.05]. Available from: http://www.scmp.com/news/china/policies-politics/article/2132001/china-has-massive-reserves-so-why-cant-it-meet-demand.

6. Blumberg KO, Walsh MP, Pera C. Low-sulfur gasoline & diesel: The key to lower vehicle emissions. World Bank, 2003.

7. ICCT. Costs and benefits of motor vehicle emission control programs in China. The International Council on Clean Transportation (ICCT), 2015.

8. ICCT. Technical and Economic Analysis of the Transition to Ultra-Low Sulfur Fuels in Brazil, China, India and Mexico. International Council on Clean Transportation (ICCT), 2012.

9. IMO. IMO sets 2020 date for ships to comply with low sulphur fuel oil requirement: International Maritime Organization (IMO), 2016 [2018.01.25]. Available from: http://www.imo.org/en/mediacentre/pressbriefings/pages/mepc-70-2020sulphur.aspx.

10. EIA. Marine Fuel Choice for Ocean-Going Vessels within Emissions Control Areas. Washington, DC: Energy Information Administration (EIA), 2015.

11. NRDC. Prevention and Control of Shipping and Port Air Emissions in China. Natural Resources Defense Council (NRDC), 2014.

12. Chinese Ministry of Transport. Annual Transportation Development Statistics Bulletin: Chinese Ministry of Transport, 2016 [2018.02.05]. Available from: http://zizhan.mot.gov.cn/zfxxgk/bnssj/zhghs/201704/t20170417_2191106.html.

13. CIA. The World Factbook. Country Comparison: Merchant Marine Central Intelligence Agency (CIA), 2018 [2018.01.31]. Available from: https://www.cia.gov/library/publications/the-world-factbook/fields/2108.html.

14. EIA. Natural Gas: U.S. Energy Information Administration (EIA), 2018 [2018.02.16]. Available from: https://www.eia.gov/naturalgas/weekly/archivenew_ngwu/2017/09_07/#tabs-prices-2.

15. Hoesly RM, Smith SJ, Feng L, Klimont Z, Janssens-Maenhout G, Pitkanen T, et al. Historical (1750–2014) anthropogenic emissions of reactive gases and aerosols from the Community Emission Data System (CEDS). Geosci Model Dev Discuss. 2017, 2017:1-41. doi: 10.5194/gmd-2017-43.

16. Loeb NG, Doelling DR, Wang HL, Su WY, Nguyen C, Corbett JG, et al. Clouds and the Earth's Radiant Energy System (CERES) Energy Balanced and Filled (EBAF) Top-of-Atmosphere (TOA) Edition-4.0 Data Product. J Climate. 2018, 31(2):895-918. doi: 10.1175/Jcli-D-17-0208.1. PubMed PMID: WOS:000425164800024.

17. Stevens B, Giorgetta M, Esch M, Mauritsen T, Crueger T, Rast S, et al. Atmospheric component of the MPI-M Earth System Model: ECHAM6. J Adv Model Earth Sy. 2013, 5(2):146-72. doi: 10.1002/jame.20015. PubMed PMID: WOS:000325277300005.

18. Stier P, Feichter J, Kinne S, Kloster S, Vignati E, Wilson J, et al. The aerosol-climate model ECHAM5-HAM. Atmos Chem Phys. 2005, 5:1125-56. PubMed PMID: WOS:000228059900001.

19. Zhang K, O'Donnell D, Kazil J, Stier P, Kinne S, Lohmann U, et al. The global aerosol-climate model ECHAM-HAM, version 2: sensitivity to improvements in process representations. Atmos Chem Phys. 2012, 12(19):8911-49. doi: 10.5194/acp-12-8911-2012. PubMed PMID: WOS:000309836800001.

20. UCAR/NCAR/CISL/TDD. The NCAR Command Language (Version 6.4.0) [Software]. Boulder, Colorado: UCAR/NCAR/CISL/TDD; 2017.

21. Du JZ, Wang KC, Wang JK, Ma Q. Contributions of surface solar radiation and precipitation to the spatiotemporal patterns of surface and air warming in China from 1960 to 2003. Atmos Chem Phys. 2017, 17(8):4931-44. doi: 10.5194/acp-17-4931-2017. PubMed PMID: WOS:000403872200001.

22. Ma Q, Wang KC, Wild M. Impact of geolocations of validation data on the evaluation of surface incident shortwave radiation from Earth System Models. J Geophys Res-Atmos. 2015, 120(14):6825-44. doi: 10.1002/2014jd022572. PubMed PMID: WOS:000359804900007.

23. Zhang XT, Liang SL, Wang GX, Yao YJ, Jiang B, Cheng J. Evaluation of the Reanalysis Surface Incident Shortwave Radiation Products from NCEP, ECMWF, GSFC, and JMA Using Satellite and Surface Observations. Remote Sens-Basel. 2016, 8(3). doi: 10.3390/rs8030225. PubMed PMID: WOS:000373627400049.

24. Shi GY, Hayasaka T, Ohmura A, Chen ZH, Wang B, Zhao JQ, et al. Data quality assessment and the long-term trend of ground solar radiation in China. Journal of Applied Meteorology and Climatology. 2008, 47(4):1006-16. doi: 10.1175/2007jamc1493.1. PubMed PMID: WOS:000255986400006.

25. Yang HB, Yang DW, Hu QF, Lv HF. Spatial variability of the trends in climatic variables across China during 1961-2010. Theor Appl Climatol. 2015, 120(3-4):773-83. doi: 10.1007/s00704-014-1208-x. PubMed PMID: WOS:000353220700030.

26. Folini D, Wild M. The effect of aerosols and sea surface temperature on China's climate in the late twentieth century from ensembles of global climate simulations. J Geophys Res-Atmos. 2015, 120(6):2261-79. doi: 10.1002/2014jd022851. PubMed PMID: WOS:000353061800006.

27. CEC. Annual Development Report of China's Power Industry 2006 (in Chinese). Beijing: China Electricity Council (CEC), 2006.

28. SEPA. A Letter to ask for comments on "11th Five-year Plan of National Environmental Protection Technology Management System Establishment (draft for comments)" (in Chinese). Beijing, China, 2007.

29. National People’s Congress. Outline of the 11th Five-Year Plan for National Economic and Social Development of the People’s Republic of China (in Chinese). Beijing, China: National People’s Congress, 2006.

30. CEC. Annual Development Report of China's Power Industry 2011 (in Chinese). Beijing: China Electricity Council (CEC), 2011.

31. MEP C. Emisson standard of air pollutants for thermal power plants Beijing: Chinese Ministry of Environmental Protection (MEP), 2011 [2017.07.17]. Available from: http://english.mep.gov.cn/standards_reports/standards/Air_Environment/Emission_standard1/201201/W020110923324406748154.pdf.

32. Wong E. On Scale of 0 to 500, Beijing's Air Quality Tops 'Crazy Bad' at 755: The New York Times, 2013 [2017.08.17]. Available from: http://www.nytimes.com/2013/01/13/science/earth/beijing-air-pollution-off-the-charts.html.

33. IAE. Energy and Air Pollution. World Energy Outlook. Special Report. Paris: International Energy Agency (IEA), 2016.

34. MEP C. Coal-fired Power Plant Ultra-low Emissions Plan: Chinese Ministry of Environmental Protection (MEP), 2015 [2017.10.25]. Available from: http://www.mep.gov.cn/gkml/hbb/bwj/201512/t20151215_319170.htm.

35. Bloomberg NEF. Dataset on solar photovoltaic projects. Bloomberg New Energy Finance, 2017.

36. JRC. Photovoltaic Geographical Information System: European Commission, Joint Research Centre (JRC), 2017 [2018.10.10]. Available from: http://re.jrc.ec.europa.eu/pvgis/apps4/pvest.php?lang=en&map=africa.

37. NDRC. Notice on Promoting the Healthy Development of Solar PV Industry through Price Leverage. National Development and Reform Commission (NDRC), 2013.

38. Sun HH, Zhi Q, Wang YB, Yao Q, Su J. China's solar photovoltaic industry development: The status quo, problems and approaches. Applied Energy. 2014, 118:221-30. doi: 10.1016/j.apenergy.2013.12.032. PubMed PMID: WOS:000332435000022.

39. Ye Qi. Annual Review of Low-Carbon Development in China: 2010. Singapore: World Scientific Publishing, 2013.

40. Pegels A. Green Industrial Policy in Emerging Countries. Abingdon, Oxon and New York, NY: Routledge, 2014.

41. NDRC. National Development and Reform Commission on the adjustment of photovoltaic power generation: National Development and Reform Commission (NDRC), 2016. Available from: http://www.ndrc.gov.cn/zcfb/zcfbtz/201612/t20161228_833049.html.
